# A Multiepitope Protein Based on Selected Synthetic Peptides Enables Effective Serological Screening of Exposure to *Mycobacterium leprae*

**DOI:** 10.1021/acsomega.5c05816

**Published:** 2025-12-22

**Authors:** Augusto César Parreiras de Jesus, Tatyane Martins Cirilo, José Bryan da Rocha Rihs, Tania Mara Pinto Dabés Guimarães, Hayana Ramos Lima, Sebastião Rodrigo Ferreira, Rocio Arreguin-Campos, Cristiane Alves da Silva Menezes, Lilian Lacerda Bueno, Bart van Grinsven, Thomas Cleij, Ana Laura Grossi de Oliveira, Ricardo Toshio Fujiwara

**Affiliations:** † Post-Graduate Program in Infectious Diseases and Tropical Medicine, School of Medicine, Federal University of Minas Gerais, Av. Prof. Alfredo Balena 190, Belo Horizonte 30130-100, Brazil; ‡ Sensor Engineering Department, Faculty of Science and Engineering, 5211 Maastricht University, Duboisdomein 30, Maastricht 6200MD, The Netherlands; § Post-Graduate Program in Parasitology, Institute of Biological Sciences, Federal University of Minas Gerais, Av. Pres. Antônio Carlos 6627, Belo Horizonte 31270-901, Brazil; ∥ Department of Clinical and Toxicological Analysis, Faculty of Pharmacy, 28114 Federal University of Minas Gerais, Av. Pres. Antônio Carlos 6627, Belo Horizonte 31270-901, Brazil; ⊥ Post-Graduate Program in Health, Environment and Biodiversity, Federal University of Southern Bahia, Av. Pres. Getúlio Vargas, 1732, Teixeira de Freitas 45996-108, Brazil

## Abstract

Early and accurate identification of individuals exposed to leprosy is essential for controlling the transmission. Our study assessed the potential of a novel recombinant multiepitope protein, designed using five synthetic *Mycobacterium leprae*-specific peptides that were selected based on their prior performance in ELISA assays. The protein was expressed in a bacterial system and further evaluated in ELISA assays for **IgG** and **IgM**. The reactivity was assessed in 357 serum samples from leprosy patients, 234 household contacts (HHC), 26 tuberculosis patients, and 61 healthy controls from different regions of Brazil. The multiepitope protein showed strong **IgG** and **IgM** responses in HHC, with AUC values above 0.80. No significant differences in antibody responses were observed between HHC of paucibacillary and multibacillary patients, for either the peptides or the novel protein. Among leprosy patients, peptides and protein responses ranged from moderate to good (AUC 0.67–0.81). These findings highlight the utility of these novel markers as promising tools for serological screening of exposure to *M. leprae*.

## Graphical abstract

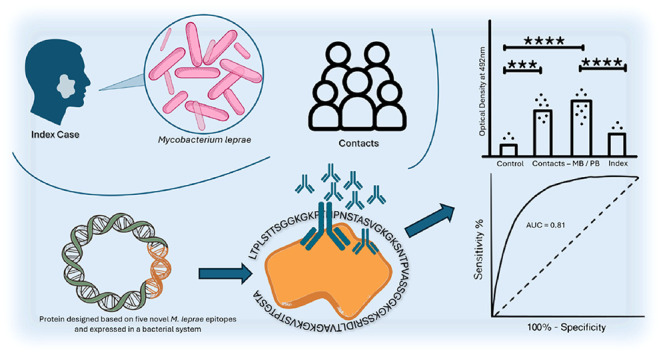

## Introduction

Leprosy persists as a neglected tropical disease, with high prevalence in countries such as India, Brazil, and Indonesia, which together accounted for approximately 80% of the 172,717 new cases reported in 2024.^1^ In these countries, certain endemic regions remain geographically isolated and distant from urban centers. Moreover, countries where the disease has been considered controlled for decades, such as the United States and Canada, are witnessing an increase in autochthonous cases.^2,3^ For example, in the United States, the total number of cases rose from 159 in 2020 to 225 in 2023.^4^ In the European Region, 27.8% of newly diagnosed cases were autochthonous in 2024.^1^ The pathogen, *Mycobacterium leprae*, has a tropism for both the skin and the peripheral nervous system cells,^5^ and it is likely transmitted through droplets from coughing and sneezing.^6^ The laboratory diagnosis of infection remains challenging due to the inability to cultivate *M. leprae* in axenic media.^7^ The most sensitive and specific tests are invasive, expensive, and require specialized personnel.^8,9^

To mitigate these challenges, the utility of immunoglobulin-based blood tests has been investigated for disease classification, monitoring of household contacts (HHC), and conducting seroprevalence studies.^10,11^ Numerous studies have been conducted to develop serological tests that detect antibodies in peripheral blood. Among these, **IgM** antibodies targeting the *M. leprae* membrane sugar antigen, phenolic glycolipid-I (PGL-1), are predominantly generated by individuals with multibacillary leprosy. Its seropositivity is strongly linked to the transmission of *M. leprae* among close contacts,^11^ being recognized as a risk factor for developing the disease in this group.^12^

Leprosy HHC represents a unique subgroup in *M. leprae* infections, whose identification is difficult. While many contacts remain asymptomatic, some eventually develop the disease.^13,14^ Notably, the levels of *M. leprae* DNA detected in some HHC can be comparable to those of paucibacillary (PB) patients.^15^ Although PGL-1 can be used to trace HHC using **IgM** detection, there is still no protein capable of detecting **IgG** in substantial quantities within this group. **IgG** responses, modulated by B-cell activators and T-cell-derived cytokines, have been proposed as potential indicators of bacterial load and treatment outcomes in leprosy.^16–18^ As a relatively simple technique, the serological detection of exposure to *M. leprae* has the potential to facilitate disease management. Early detection of exposure to *M. leprae* is crucial for preventing complications and interrupting the chain of transmission.

Synthetic peptides, derived from epitopes identified through the analysis of patient antibody reactivity, have emerged as promising candidates for the development of serological diagnostic tools for a variety of neglected tropical diseases.^19^ B-cell epitopes correspond to specific portions of a molecule to which certain antibodies bind, also known as antigenic determinants. The spatial proximity of amino acid residues, influenced by the protein’s conformation, determines the classification of an epitope as either conformational or linear.^20^ The use of these epitopes in the development of diagnostic tools, facilitated by advances in bioinformatics and molecular biology, offers significant advantages, including a reduction of time and financial resources, as well as a decreased need for animal experimentation.^21–23^ Another notable benefit is the potential for engineering multiepitope recombinant platforms, which can simultaneously accommodate multiple antigenic epitopes, a feature that distinguishes them from natural proteins.^24–26^

In recent years, several studies have demonstrated the potential of epitope-based synthetic peptides for serodiagnosis of leprosy.^19,27,28^ Our research group previously selected synthetic peptides with promising performance for detecting exposure to *M. leprae* among HHC of leprosy patients.^29^ Notably, studies have shown that antibody levels detected by conventional serological biomarkers are higher in HHC of MB patients compared to those of PB patients.^30,31^ Therefore, in the present study, we aimed to downselect the previously identified peptides to investigate potential differences in antibody responses among HHC of patients with either PB or MB forms of the disease. In parallel, we sought to design and evaluate a novel multiepitope protein based on these selected markers and to validate the individual peptides and the recombinant protein for their ability to detect *M. leprae* exposure by assessing immunoglobulin responses in household contacts compared to other groups. Identifying such markers may provide valuable tools for epidemiological surveillance and strengthen public health strategies aimed at controlling bacterial transmission.

## Results

Figure 1 presents the geographic distribution of sampling sites located in the Brazilian states of Minas Gerais, Bahia, and Sergipe.

**Figure 1:**
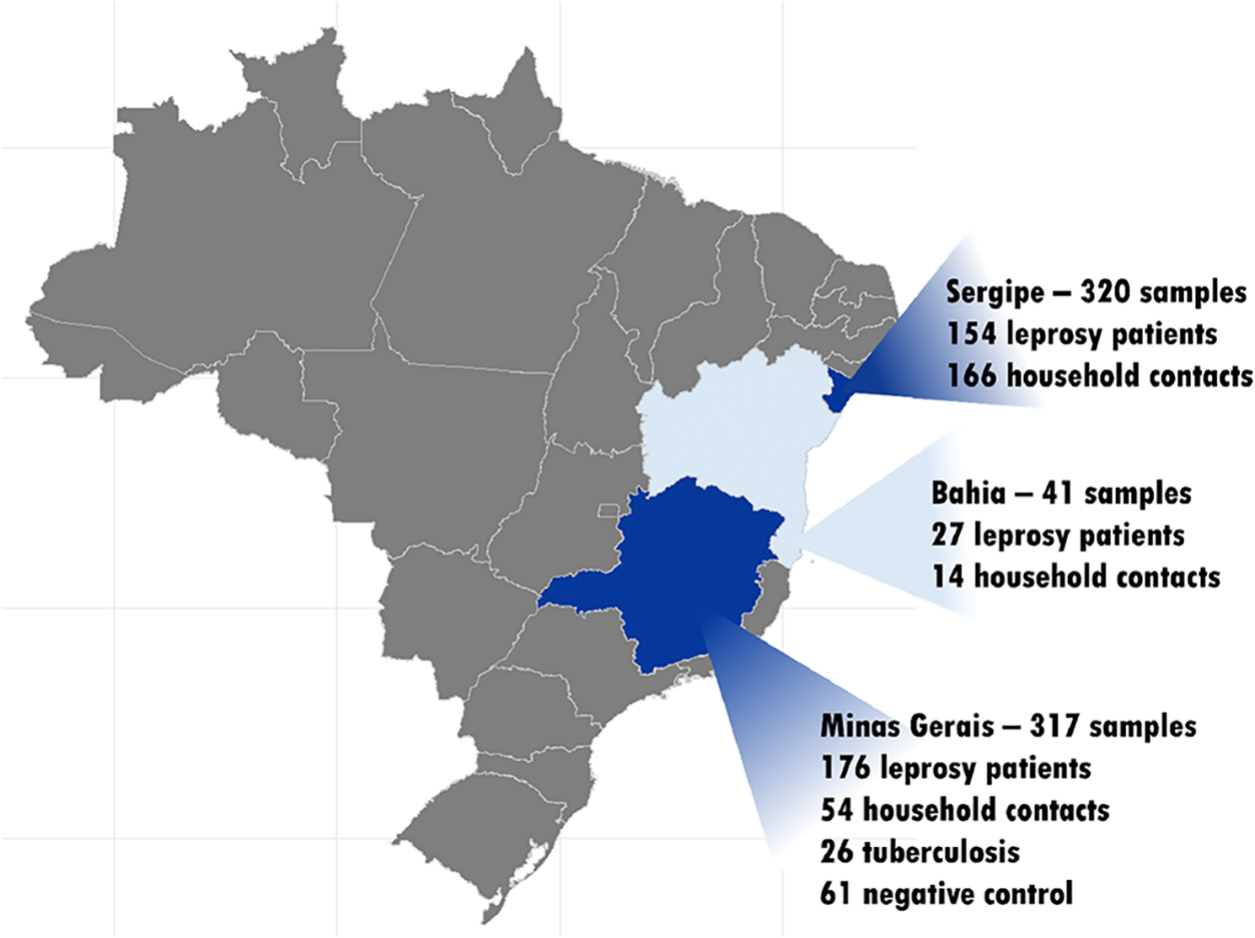
Map of Brazil highlighting the states of Minas Gerais (*n* = 317), Bahia (*n* = 41), and Sergipe (*n* = 320). These samples are part of the Biorepository at the Laboratory of Immunobiology and Control of Parasites, Biological Science Institute, Federal University of Minas Gerais.

### *M. leprae* Synthetic Peptides Were More Strongly Recognized by Antibodies from Household Leprosy Contacts

The peptides used in this study, designated as **PEP1** (LTPLSTTSG), **PEP2** (PTPNSTASV), **PEP3** (SNTPVASSG), **PEP4** (SSRIDLTVA), and **PEP5** (VSTPTGSTA), were derived from a previous work conducted by our group, as described in de Jesus et al.^29^ The peptide results presented in this study integrate previously published data with newly included samples to evaluate their ability to discriminate **IgG** levels among individuals within the household contacts group and to determine whether their reactivity profiles remain consistent in a larger cohort. Based on the **IgG** seropositivity, Figure 2 shows that the tested peptides, **PEP1**, **PEP2**, **PEP3**, **PEP4**, and **PEP5**, were effective in distinguishing healthy individuals (Negative Control) from leprosy patients, as well as household contacts of paucibacillary (HHC-PB) and multibacillary (HHC-MB) leprosy patients, with significant differences observed across all groups. **PEP3**, **PEP4**, and **PEP5** were more effective in differentiating Negative Control from the leprosy group (*p* ≤ 0.0001). Higher levels of **IgG** were observed in household contacts of both PB and MB patients compared with leprosy patients for all peptides (*p* ≤ 0.0001). However, no significant difference was observed between the household contacts of paucibacillary (HHC-PB) and multibacillary (HHC-MB) leprosy patients. Exact *p*-values for each peptide are as follows: **PEP1**: *p* (negative control vs leprosy patients) = 0.0027; *p* (HHC-MB vs HHC-PB) = 0.3258. **PEP2**: *p* negative control vs leprosy patients = 0.0019; *p* (HHC-MB vs HHC-PB) > 0.9999. **PEP3**: *p* (HHC-MB vs HHC-PB) > 0.9999 **PEP4**: *p* (HHC-MB vs HHC-PB) > 0.9999. **PEP5**: *p* (HHC-MB vs HHC-PB) = 0.8265.

**Figure 2:**
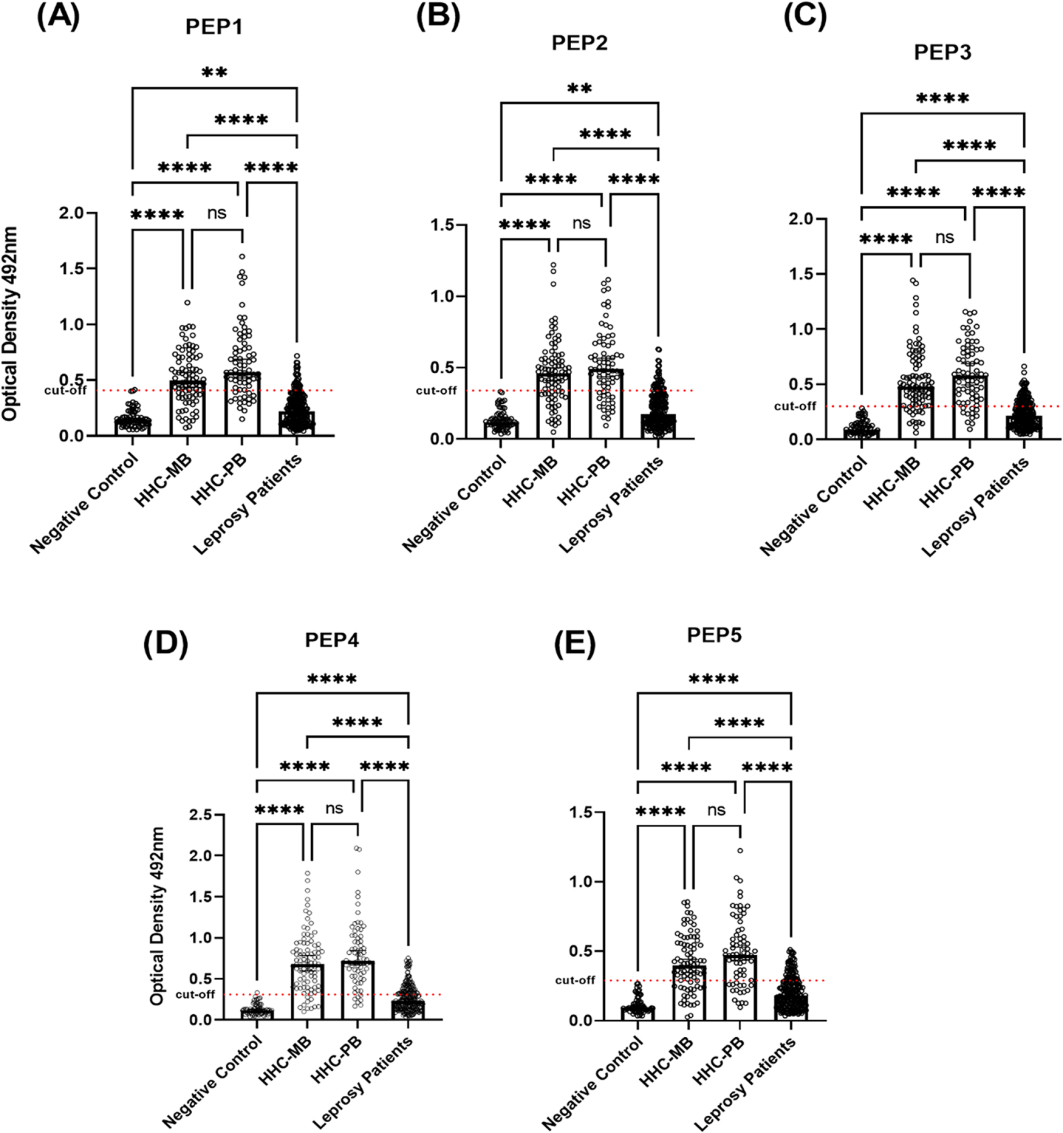
**IgG** antibody reactivity to the five synthetic peptides among the different study groups. The figure shows the median optical density (OD) values of **IgG** antibodies for each peptide in healthy controls, household contacts (HHC) of PB and MB leprosy patients, and leprosy patients. (A) Peptide **PEP1**, (B) Peptide **PEP2**, (C) Peptide **PEP3**, (D) Peptide **PEP4**, and (E) Peptide **PEP5**. Statistical analysis was performed using the Kruskal–Wallis test, followed by Dunn’s multiple comparison tests. The asterisks indicate statistical significance. ** *p* ≤ 0.01; **** *p* ≤ 0.0001.

In addition, Table 1 presents the OD results for **IgG** serology against five peptides (**PEP1**-**PEP5**) across the different study groups. For each peptide, the table provides the median OD values, the corresponding interquartile range (IQR), and cutoff values.

**Table 1:** Median Optical Density (OD) Values, Interquartile Range (IQR), and Cutoff Values for **IgG** Serology against Peptides **PEP1**–**PEP5** in Negative Controls, Household Contacts of MB and PB Leprosy Patients, and Leprosy Patients

peptides	negative control (IQR)	HHC-MB (IQR)	HHC-PB (IQR)	leprosy patients (IQR)	cutoff
**PEP1**	0.1407 (0.1013–0.2143)	0.4939 (0.3514–0.7058)	0.5715 (0.4358–0.8192)	0.2196 (0.1251–0.3306)	0.4105
**PEP2**	0.1192 (0.0907–0.1669)	0.4604 (0.3167–0.5664)	0.4905 (0.3411–0.6685)	0.1758 (0.1109–0.3012)	0.3419
**PEP3**	0.0902 (0.0639–0.1460)	0.4808 (0.3559–0.7182)	0.5776 (0.3768–0.8115)	0.2146 (0.1276–0.3161)	0.3036
**PEP4**	0.1181 (0.0824–0.1646)	0.6782 (0.4114–0.9083)	0.7176 (0.5485–1.007)	0.2306 (0.1455–0.3546)	0.3226
**PEP5**	0.0925 (0.0778–0.1332)	0.3977 (0.2551–0.5597)	0.4735 (0.3171–0.6415)	0.1795 (0.1098–0.2802)	0.2861

### Design and Characterization of a Protein for Identifying Household Contacts

Given the **IgG** seropositivity results gathered from assays using the synthetic peptides derived from *M. leprae* proteins, which demonstrated a distinct ability to identify individuals, particularly household contacts of leprosy patients,^29^ a novel multiepitope protein was designed with the following molecular formula: C_242_H_420_N_74_O_87_, with a molecular weight of 5.76 kDa (Figure 3A). The protein is likely predominantly in a coil shape (Figure 3B), while the region corresponding to **PEP4** appears to form a β-strand conformation. None of the protein’s residues are predicted to be highly exposed on the surface; however, the highest solvent accessibility scores are associated with **PEP1** and **PEP5** (Figure 3C). Approximately 75% of the residues are predicted to be structurally stable, particularly those within the **PEP4** region (Figure 3D).

**Figure 3:**
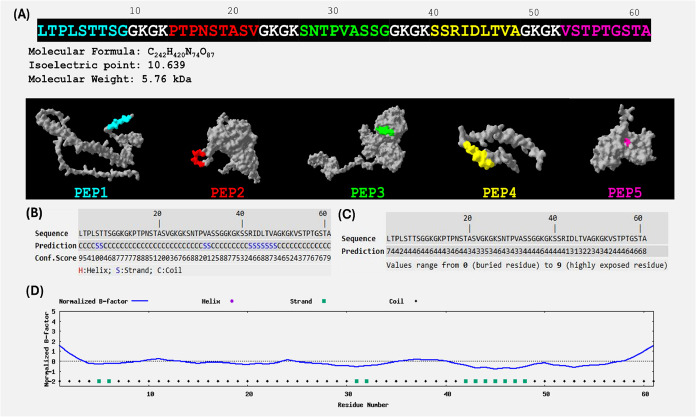
Sequence and structural characterization of the designed multiepitope protein. (A) The amino acid sequence of the novel protein, along with its molecular formula, weight, and isoelectric point. The contributing peptides are as follows: Epitope 1 (**PEP1**): Derived from WP_010907635.1 septum formation initiator family protein (positions 1–9). Epitope 2 (**PEP2**): Derived from the WP_162618330.1 ABC transporter family substrate-binding protein (positions 14–22). Epitope 3 (**PEP3**): Derived from WP_010908856,1 type I polyketide synthase protein (positions 27–35). Epitope 4 (**PEP4**): Derived from WP_162618330.1 ABC transporter family substrate-binding protein (positions 40–49). Epitope 5 (**PEP5**): Derived from WP_010908290.1 NAD kinase (positions 54–62). (B) Predicted Secondary Structure: sequence-based prediction of the secondary structure. Higher scores indicate greater confidence in predicted structure. (C) Predicted solvent accessibility. (D) Predicted normalized B-factor: negative values indicate that the residue is relatively more stable within the structure. This figure was generated using Swiss-PDBViewer, Protpi, and I-TASSER.

The protein containing the five peptides showed promising serological performance. The HHC group exhibited the highest antibody reactivity, with statistically significant differences compared to those of the other tested groups for both **IgM** and **IgG** responses. The leprosy patient group displayed lower median antibody levels of both immunoglobulins than the HHC group but higher levels than the negative controls. No statistically significant differences were observed between the two control groups (negative controls and tuberculosis patients) for **IgM** and **IgG** (Figure 4). Median OD values and exact *p* values are presented below:

**Figure 4:**
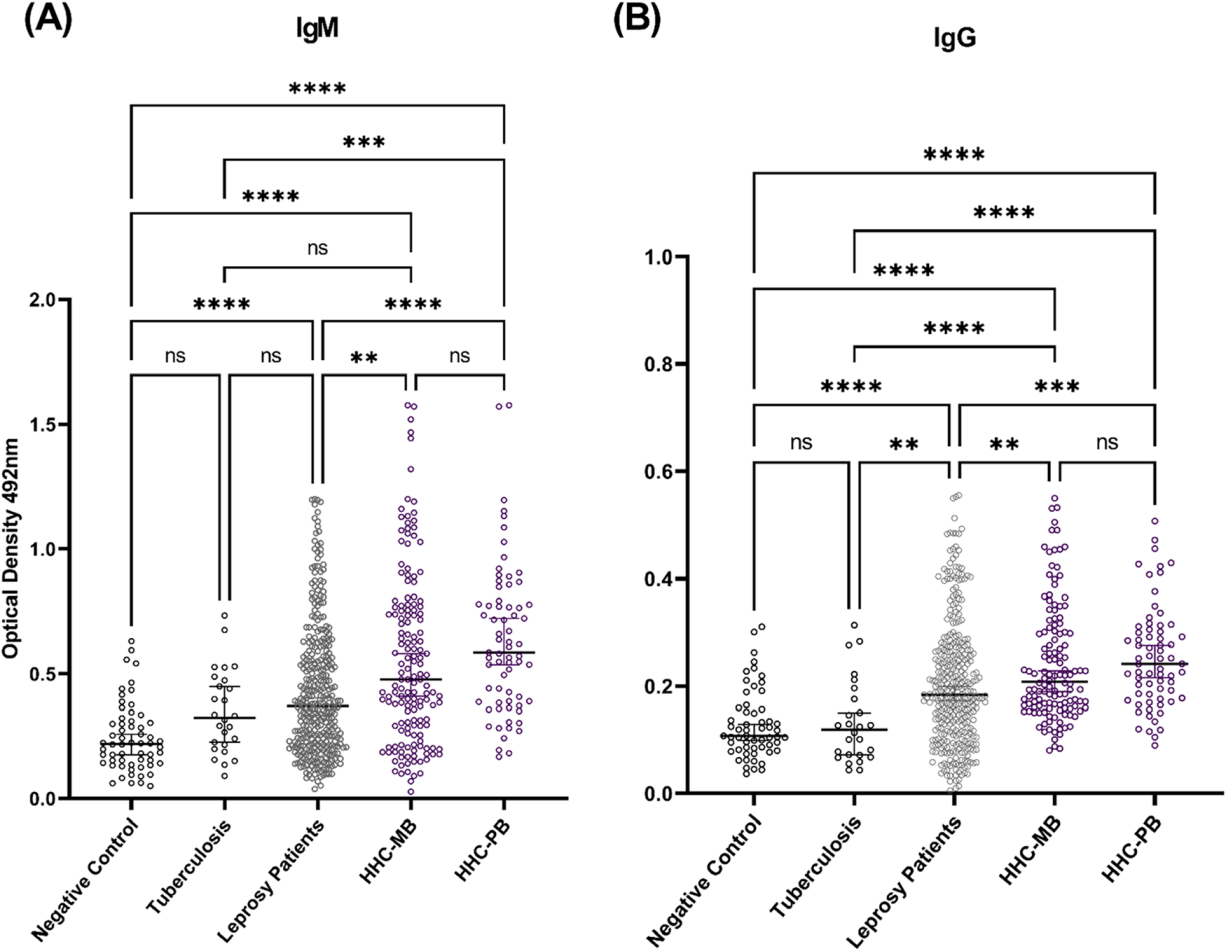
**IgM** and **IgG** antibody reactivity to the designed protein across different study groups. The figure shows the median optical density (OD) values of (A) **IgM** and (B) **IgG** antibodies for the protein in healthy controls, tuberculosis patients, household contacts (HHC) of PB and MB leprosy patients, and leprosy patients. Statistical analysis was performed using the Kruskal–Wallis and ANOVA tests, followed by Dunn’s multiple comparison tests. The asterisks indicate statistical significance. ** *p* ≤ 0.01; *** *p* ≤ 0.001; **** *p* ≤ 0.0001.

#### IgM

Negative Control: 0.2202; Tuberculosis: 0.3245; Leprosy Patients: 0.3703; Household Contacts of MB patients (HHC-MB): 0.4770; Household Contacts of PB patients (HHC-PB): 0.5856. Statistical comparisons: **p = 0.0022, ***p = 0.0005, ****p < 0.0001; p (negative control vs tuberculosis) = 0.2785; *p* (tuberculosis vs leprosy patients) > 0.9999; *p* (tuberculosis vs HHC-MB) = 0.0590; *p* (HHC-MB vs HHC-PB) = 0.1523.

#### IgG

Negative Control: 0.1079; Tuberculosis: 0.1193; Leprosy Patients: 0.1834; HHC-MB: 0.2080; HHC-PB: 0.2413. Statistical comparisons: ***p = 0.0004, ****p < 0.0001; *p* (negative control vs tuberculosis) > 0.9999; *p* (tuberculosis vs leprosy patients) = 0.0088; *p* (leprosy patients vs HHC-MB) = 0.0036; *p* (HHC-MB vs HHC-PB) > 0.9999.

The receiver operating characteristic (ROC) curves revealed a strong discriminatory performance, particularly for **PEP3**, **PEP4**, and **PEP5**, which exhibited the highest area under the curve (AUC) values. Among these, **PEP3** showed the most promising results, with values around or exceeding 0.8 when comparing healthy controls with both household contacts and leprosy patients. In contrast, the multiepitope protein displayed slightly lower performance compared to the individual peptides (Figure 5). Considering that no statistically significant differences were observed between the HHC-MB and HHC-PB groups in the OD values (Figures 2 and 4), these groups were combined for the ROC analyses.

**Figure 5:**
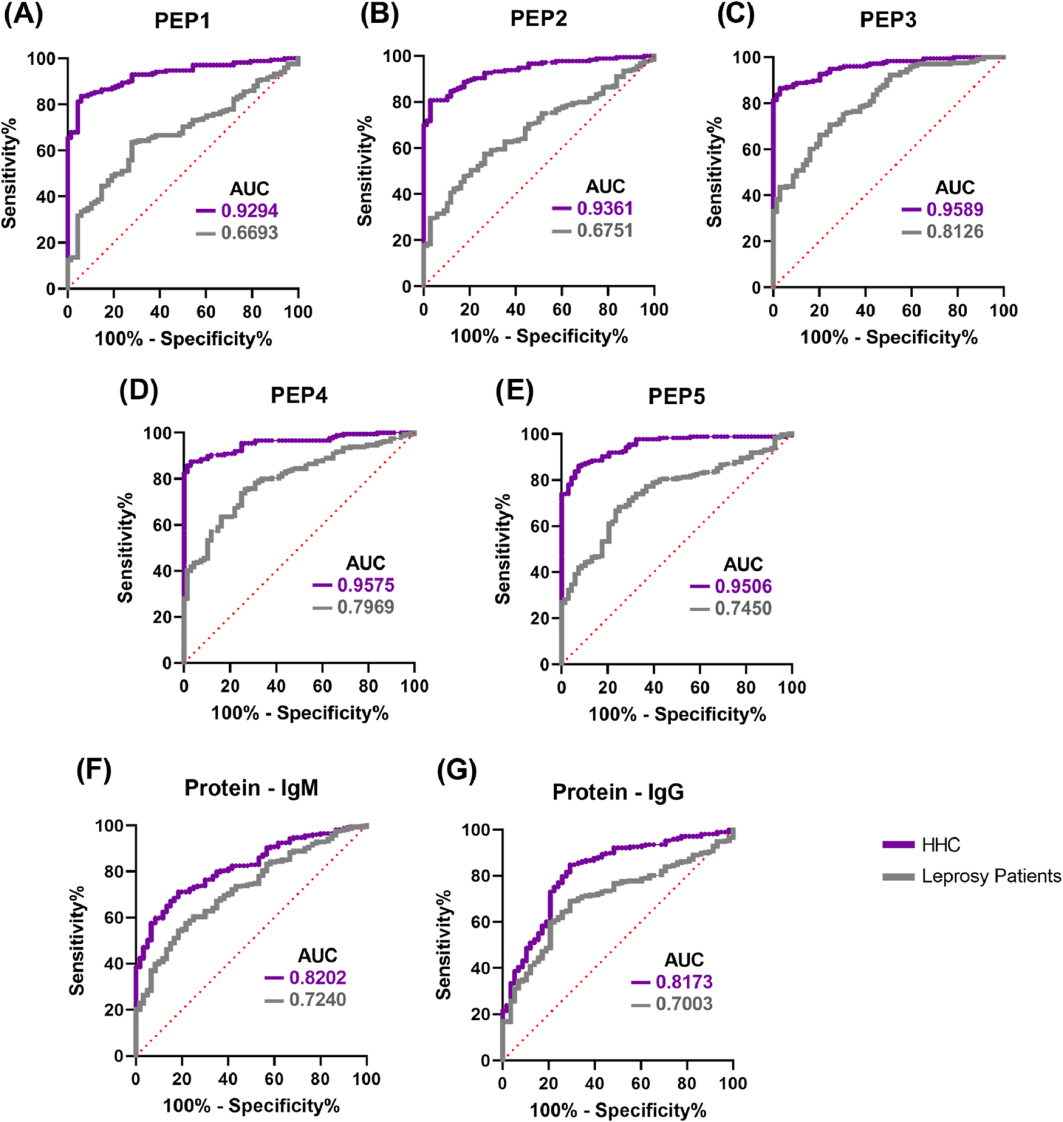
Receiver operating characteristic (ROC) curves for the diagnostic performance of the evaluated markers. The ROC curves show the sensitivity and specificity in distinguishing HHC from healthy controls and leprosy patients from healthy controls, using (A) **PEP1**, (B) **PEP2**, (C) **PEP3**, (D) **PEP4**, and (E) **PEP5** to detect **IgG**, and the designed protein to detect (F) **IgM** and (G) **IgG**. The dashed red diagonal line represents random classification (AUC = 0.50). All comparisons showed statistically significant discrimination (*p* < 0.0001).

Detailed AUC estimates for each peptide and the protein are presented below. Values are expressed as the standard error (SE) and 95% confidence interval (CI):

**PEP1** – HHC: SE = 0.01593; 95% CI: 0.8982–0.9606; Patients: SE = 0.03288; 95% CI: 0.6048–0.7337. **PEP2** – HHC: SE = 0.01461; 95% CI: 0.9074–0.9647; Patients: SE = 0.03257; 95% CI: 0.6112–0.7389. **PEP3** – HHC: SE = 0.01088; 95% CI: 0.9375–0.9802; Patients: SE = 0.02716; 95% CI: 0.7594–0.8658. **PEP4** – HHC: SE = 0.01154; 95% CI: 0.9349–0.9801; Patients: SE = 0.02709; 95% CI: 0.7438–0.8500. **PEP5** – HHC: SE = 0.01288; 95% CI: 0.9254–0.9758; Patients: SE = 0.02991; 95% CI: 0.6864–0.8036. **Protein IgM** – HHC: SE = 0.02601; 95% CI: 0.7692–0.8711; Patients: SE = 0.03179; 95% CI: 0.6617–0.7863. **Protein IgG** – HHC: SE = 0.03117; 95% CI: 0.7562–0.8784; Patients: SE = 0.03187; 95% CI: 0.6378–0.7627.

## Discussion

The diagnosis of leprosy is primarily based on the identification of neural and dermatological clinical signs and symptoms, which can take a long time to appear after exposure to the *Mycobacterium leprae*.^32,33^ However, developing reliable diagnostic tools remains crucial for improving disease management and reducing transmission. In this context, enhanced methods for screening *M. leprae* exposure are essential to limiting the establishment of leprosy. Since the late 1990s, several genomics and proteomics studies have been conducted on *M. leprae*, contributing to the comprehensive description of the bacterium’s genome and the identification of various potentially specific proteins. By leveraging information available in various biological databases, it is now possible to predict specific immunogenic regions of this microorganism.^34–36^

An antigen-binding site is much smaller than a large macromolecule, meaning that only a fragment of a protein can stimulate a single B cell. However, there is no definitive agreement about the exact size of the antigenic determinant recognized by antibodies. Existing data suggest that short peptides (7–10 amino acids) could serve as such determinants.^37,38^ Our group predicted 9- and 15-mer epitopes as promising candidates for enhancing leprosy diagnostic tools. Interestingly, these epitopes, synthesized as peptides, detected strong antibody responses in household contacts, even surpassing some commonly used antigens in current serological tests.^29^ Encouraged by these results, we focused our further investigation on the peptides that showed the most significant responses. In addition to the results presented in this study (Figure 2), it is evident that the predicted peptides successfully exhibited significant antibody responses in individuals with a history of *M. leprae* exposure, specifically in household contacts of leprosy patients, individuals exposed to the bacterium who have not yet developed the disease. Our findings strongly support the hypothesis that peptides of 7–10 amino acids are likely to function as antigenic determinants, with the characteristics of our 9-mer peptides reinforcing this conclusion. Other groups have also demonstrated the relevance of synthetic peptides, either individually or in combination, for the serological diagnosis of leprosy, although the identification of household contacts remains less explored and has so far been addressed in smaller cohorts compared to ours. However, these peptides have shown high accuracy for PB patients.^19,28,39^

Interestingly, we observed that these peptides were able to detect comparable antibody levels in both household contacts of patients with PB or MB leprosy. This finding contrasts with previous studies, which reported weaker **IgG** responses in contacts of PB patients compared to those of MB patients.^30,31^ This unique characteristic of our peptides offers an opportunity to refine the monitoring strategies for individuals at risk of developing leprosy. By identifying individuals who present antibody responses even in the absence of apparent clinical symptoms, we could develop more sensitive diagnostic tools for early detection, particularly in endemic regions. Furthermore, this approach could aid in identifying asymptomatic individuals or those in the early stages of infection, enabling timely interventions and better-targeted prevention programs.

Genetic variability influences antibody recognition and immunoassay performance. Polymorphisms in protein-coding regions or in glycosylation pathways can alter epitope structure, while variation in HLA-II alleles affects which antigenic peptides are presented, shaping individual antibody specificity.^40,41^ For instance, Teles et al. reported associations between seropositivity for NDO-LID and PGL-1 and specific HLA alleles.^42^ Our peptides might also be linked to genetic features, although this requires further research. Another hypothesis is that antigen presentation processes might become more effective as bacterial load increases in lesions, or that multidrug therapy (MDT)-induced bacilli clearance might enhance some antigen presentation.^43^ Additionally, immune recognition of the proteins containing our peptides might differ due to specific genetic or cellular responses unique to individuals who remain as contacts, a phenomenon that has yet to be fully understood. These factors can lead to differences in immunoreactivity between individuals, impacting assay sensitivity and specificity, and highlight the need to consider epitope selection when designing diagnostic assays.

Based on these findings, we designed a multiepitope protein incorporating these five peptides (Figure 3) to improve detection capabilities by increasing the number of immunogenic sites within a single molecule. Recent studies have shown that the use of synthetic peptides in diagnostic tools can achieve higher specificity, improving from 70 to 90% with conventional commercial methods to nearly 97% with peptide-based approaches. This highlights their ability to reduce cross-reactivity, whether the epitopes are used individually or combined.^44,45^ With this multiepitope protein, **IgG** detection outperformed **IgM** in demonstrating statistical differences between groups with a history of *M. leprae* exposure (patients and contacts) and those without known exposure (Figure 4, Tables 2 and 3). In the HHC groups, the **IgG** responses detected were approximately twice as high as those in the negative and tuberculosis control groups. However, the overall efficacy of the protein was found to be similar for both immunoglobulins (Figure 5). Previous studies have explored the potential of **IgG** antibodies against markers such as Leprosy Infectious Disease Research Institute (IDRI) Diagnostic-1 (LID-1) and NDO-LID for diagnosing *M. leprae* infections.^46–48^ Nevertheless, their diagnostic value remains limited for patients and is still under investigation for household contacts.

**Table 2:** Sensitivity, Specificity, and Accuracy (%) of ELISA Assays Using Individual Peptides (**PEP1–PEP5**) and the Multiepitope Protein (**IgM and IgG**) Evaluated in Serum Samples from Household Contacts of Individuals Diagnosed with Leprosy^a^

	cutoff	AUC	sensitivity (%)	95 CI (%)	specificity (%)	95 CI (%)
**PEP1**	>0.2444	0.9294	85.96	79.97 to 90.38	85.29	75.00 to 91.81
**PEP2**	>0.2218	0.9361	85.31	79.35 to 89.77	86.76	76.72 to 92.88
**PEP3**	>0.2123	0.9589	88.76	83.28 to 92.61	89.86	80.51 to 95.00
**PEP4**	>0.2193	0.9575	89.77	84.41 to 93.43	89.71	80.24 to 94.92
**PEP5**	>0.2048	0.9506	87.28	81.50 to 91.45	89.71	80.24 to 94.92
**Protein IgM**	>0.3059	0.8202	73.36	67.29 to 78.67	75.00	62.77 to 84.22
**Protein IgG**	>0.1602	0.8173	75.23	69.10 to 80.49	77.59	65.34 to 86.41

a AUC = area under the curve; CI = confidence interval; values are related to the responses in the negative sera.

**Table 3:** Sensitivity, Specificity, and Accuracy (%) of ELISA Assays Using Individual Peptides (**PEP1–PEP5**) and the Multiepitope Protein (**IgM and IgG**) Evaluated in Serum Samples from Leprosy Patients under Treatment^a^

	cutoff	AUC	sensitivity (%)	95 CI (%)	specificity (%)	95 CI (%)
**PEP1**	>0.1678	0.6693	64.26	58.13 to 69.95	69.12	57.36 to 78.83
**PEP2**	>0.1383	0.6751	62.70	56.48 to 68.53	64.71	52.84 to 75.00
**PEP3**	>0.1397	0.8126	71.14	65.19 to 76.44	72.46	60.95 to 81.61
**PEP4**	>0.1436	0.7969	73.77	67.91 to 78.89	75.00	63.56 to 83.76
**PEP5**	>0.1217	0.7450	71.14	65.19 to 76.44	70.59	58.89 to 80.08
**Protein IgM**	>0.2748	0.7240	64.22	59.00 to 69.13	66.67	54.06 to 77.27
**Protein IgG**	>0.1370	0.7003	68.93	63.81 to 73.63	70.69	57.99 to 80.82

a AUC = area under the curve; CI = confidence interval; values are related to the responses in the negative sera.

In this context, small synthetic peptides with their linear structure may facilitate efficient antibody binding. In contrast, proteins expressed in bacterial systems may encounter challenges such as post-translational modifications, incorrect or incomplete folding, aggregation, or contamination, such as endotoxins.^49–51^ These factors can affect protein folding, stability, and solubility. Despite these limitations, bacterial expression offers key advantages: rapid and inexpensive growth, high-yield, large-scale production, widely available vectors and tools, fast expression, and ease of genetic manipulation. These strengths make bacterial systems a highly attractive option compared to yeast, insect, or mammalian expression platforms.^50,52,53^ Our findings indicate that the protein’s overall performance was lower than that of the individual peptides. This reduction may be due to structural factors, such as key peptides like **PEP3** and **PEP4** being obscured within the protein, thereby limiting antibody access and binding. In the predicted secondary structure of our protein (Figure 3B), **PEP4** appears as a strand and seems to be a relatively stable sequence within the structure (Figure 3D). However, its residues have a low predicted solvent accessibility score (Figure 3C), suggesting a limited exposure to antibodies in the samples. B cells recognize solvent-exposed antigens through their B-cell receptors (BCRs) and, upon activation, differentiate into plasma cells that secrete soluble immunoglobulins.^54^ Furthermore, steric hindrance can also hinder antibody binding, particularly in multimeric protein contexts. This implies that while the multiepitope construct successfully integrates several immunogenic regions, the spatial arrangement of these epitopes within a folded protein may restrict their availability for antibody recognition. This can occur due to limited spatial access caused by neighboring molecules or the presence of other antibodies bound to adjacent sites. In other words, steric hindrance arising from intra- or intermolecular interactions could physically block access to certain epitopes, particularly when adjacent regions fold closely together.^55^ Unlike linear peptides, natural antigens such as folded proteins are more appropriately considered as two-dimensional lattice surfaces rather than one-dimensional molecules. As a result, the effects of steric hindrance on the binding of ligands to such surfaces are therefore more complex.^56^ Additionally, partial misfolding during bacterial expression may lead to conformations that mask hydrophilic residues or form aggregates, further reducing binding efficiency.^57^ Such effects are consistent with the slightly lower performance of the recombinant protein compared with free linear peptides, which remain fully accessible to antibodies in solution.^58^ Moreover, the antigen valency of our protein may also not be optimal, influencing antibody binding efficiency and the overall immunoreactivity of the construct.^59^

Several strategies could be considered to improve the structural performance of the protein. For instance, fusion to stabilizing tags or small scaffolds may further enhance expression and maintain proper folding,^60^ while the introduction of additional flexible linkers between epitopes could reduce steric hindrance and improve solubility, potentially without altering the functional epitopes.^61^ Designing alternative multiepitope proteins, such as those using only **PEP3** and **PEP4**, could also be advantageous.^26^ Moreover, combining our peptides with other antigens, such as PGL-1, may enhance *M. leprae* exposure detection and help differentiate between exposure and active disease. Despite assay optimizations for the protein, challenges persist, particularly given the protein sequence’s 5-fold increase in size and expression in a biological system.

Despite its lower performance compared to the individual peptides, our protein demonstrated a remarkable ability to detect elevated antibody levels in leprosy contacts relative to other groups, with positive predictive values of 85.2 and 88.7% for **IgM** and **IgG**, respectively (Table S1). Importantly, this outcome indicates that the protein components possess a distinctive feature because contacts are at increased risk of developing leprosy and may contribute to transmission. Although less contagious, their *M. leprae* DNA levels are comparable to those of PB leprosy patients.^15^

In this regard, active case detection among household, social, and school contacts has proven to be a highly effective strategy for identifying new cases of leprosy, with the highest incidence observed in both high- and low-endemicity areas.^62^ Moreover, longitudinal follow-up of seropositive contacts, particularly for PGL-1, demonstrates a high likelihood of detecting new active cases.^63^ Consequently, monitoring antibody levels against our identified peptides offers promising potential as accessible, efficient, and cost-effective biomarkers with the added benefit of predicting disease progression. This approach could enhance early detection and interventions, particularly in high-risk populations, as well as in regions where the disease was not previously endemic but where an increasing number of autochthonous cases has been observed, such as in the United States and Canada.^2,3^ Furthermore, as emerging evidence suggests potential zoonotic transmission routes for *M. leprae*, tracing exposed individuals becomes even more critical.^64^ A recent report described a leprosy case in an individual with no history of travel to endemic areas or known contact with patients, but who reported indirect exposure to armadillos.^65^ Some other authors also provided evidence of exposure associated with hunting or consumption of these animals.^66–68^ Thus, optimizing detection methods and accurately mapping vulnerable populations are essential steps toward more effective leprosy control strategies.

Although only a minority of native antigens contain linear B-cell epitopes^69^ and some synthetic peptides may exhibit low reactivity against specific antibodies,^23^ our study shows that they can serve as effective alternatives for antibody detection in household contacts of leprosy, as it has been demonstrated for other diseases.^70–72^ Linear B-cell epitopes can readily replace whole antigens for immunization and antibody production.^20^ Since our peptides elicit antibody responses in asymptomatic individuals, their potential as vaccine candidates could also be explored in future studies.

Our study has some limitations, including sampling restricted to a single country and the absence of longitudinally collected samples, particularly from contacts. However, the geographic limitation is mitigated by Brazil’s remarkable genetic diversity, with predominantly European ancestry in Minas Gerais and predominantly African ancestry in Sergipe and Bahia.^73^ Nevertheless, despite this genetic diversity, it will be important to evaluate our markers in populations from other countries heavily affected by leprosy to ensure their broader applicability. Unfortunately, we lack information about whether the contacts later developed the disease. In future studies, it will be important to evaluate our markers using serum samples from contacts and patients in a longitudinal approach, allowing a more comprehensive assessment of their predictive value and potential applications in early diagnosis and disease monitoring. However, our novel multiepitope protein or its peptides show promise for facilitating the screening of *Mycobacterium leprae* exposure in household contacts through **IgG** detection in serum. These findings highlight the potential of small synthetic peptides as highly accurate biomarkers. Further investigation is required to elucidate the mechanisms underlying their immune system stimulation pathways. Moving forward, integrating these markers into alternative immunological approaches and diagnostic platforms, such as immunochromatography strips, electrochemical biosensors, or sequence conjugations, may enhance sensitivity and specificity, ultimately improving screening and disease control strategies. In this context, the peptides or multiepitope protein could be immobilized on lateral flow membranes or biosensor surfaces to enable rapid, point-of-care detection without the need for specialized laboratory infrastructure, facilitating large-scale screening and timely intervention in endemic regions.

## Material and Methods

### Human Serum Samples

Serum samples from 678 individuals in Brazil, collected from two distinct geographical regions of Brazil, Southeast (Minas Gerais state) and Northeast (Bahia and Sergipe states), were distributed as follows: 357 leprosy patients, 234 household contacts, 26 tuberculosis patients, and 61 healthy control subjects from both endemic and nonendemic areas (Figure 1). The samples were given to the Biorepository at the Laboratory of Immunobiology and Control of Parasites (LICP-UFMG). All individuals included in this study were anonymized and provided written informed consent before sample collection. Leprosy diagnosis was performed by specialist physicians at their respective healthcare facilities using the WHO operational classification: paucibacillary and multibacillary. Tuberculosis patients were diagnosed based on clinical criteria. The study protocol was approved by the Ethical Committee of the Federal University of Minas Gerais (COEP: #11884919.4.0000.5149) and conducted in accordance with the guidelines of the National Health Council of Brazil.

### Protein Designing, Bacterial Transformation, and Protein Expression

Based on the results of the ELISA tests for peptides, a multiepitope protein was designed using five novel peptides. The design considered the expression vector pET28a­(+)-TEV (GenScript, USA), with glycine (G) and lysine (K) residues strategically introduced as spacers between the epitopes. The resulting amino acid sequence of the protein is as follows: LTPLSTTSGGKGKPTPNSTASVGKGKSNTPVASSGGKGKSSRIDLTVAGKGKVSTPTGSTA. To facilitate the subcloning of the gene into the expression vector, restriction enzyme sites, NdeI and *Hin*dIII, were incorporated at the 5′ and 3′ ends, respectively. Electrocompetent *Escherichia coli* C43 cells (Phoneutria, Brazil) were transformed by electroporation using a 1.8 kV electric pulse with a MicroPulser Electroporation Apparatus (Bio-Rad Laboratories, USA) and the recombinant plasmid pET28a­(+)-TEV/hans. The gene insertion was confirmed by colony PCR using T7 universal primers (Macrogen, South Korea). Protein expression in the transformed *E. coli* cells was induced by adding 1 mM Isopropyl-ß-D-Thiogalactopyranoside (IPTG) (Thermo Fisher Scientific, Brazil) and incubating for 3 h at 37 °C with shaking at 180 rpm, using a refrigerated incubator with shaker TE-421 (Tecnal, BR). Samples from both soluble and insoluble fractions were analyzed by sodium dodecyl sulfate-polyacrylamide gel electrophoresis (SDS-PAGE) to determine the solubility of the recombinant protein. Additionally, cell extract fractions were analyzed by SDS-PAGE to compare samples taken before (0 h) and after (3 h) the addition of IPTG to induce gene expression in bacterial systems.

The bacterial cells were resuspended in buffer A (20 mM NaH_2_PO_4_; 500 mM NaCl; 30 mM imidazole; 8 M urea) at a volume equivalent to 10% of the initial culture volume. The cells were lysed with lysozyme (100 μg/mL) for 30 min, followed by five cycles of mechanical lysis using the EmulsiFlex-C3 homogenizer (AVESTIN, Canada), with pressure peaks between 15,000 and 20,000 psi. The suspension was then centrifuged at 10,000*g* for 1 h at 4 °C, and the supernatant was filtered through a 0.45 μm filter (Jet Biofil, China) before purification. The pET28a-TEV expression vector contains a sequence encoding a histidine tag in the N-terminal region, facilitating protein purification. The recombinant protein was purified using Ni^2+^ affinity chromatography with a His-Trap HP column (GE Healthcare Life Science, Brazil) and an ÄKTA Prime Plus system (GE Healthcare Life Science, Brazil). The protein was eluted using a linear gradient from 0 to 100% buffer B (20 mM NaH_2_PO_4_; 500 mM NaCl; 500 mM Imidazole). To assess the purity and yield of the purified recombinant protein, the fractions were analyzed by 16% SDS-PAGE, and the final protein concentration was determined using the BCA Protein Assay (Thermo Fisher Scientific, USA), following the manufacturer’s instructions. Protein concentration was determined by measuring absorbance at 562 nm using an automated microplate ELISA reader (VersaMax, Molecular Devices, USA) with SoftMax Pro 4.8 software. The results were compared to a standard curve generated from known concentrations of bovine serum albumin (BSA), measured in mg/mL.

### Immunoglobulin Detection by Enzyme-Linked Immunosorbent Assay (ELISA)

ELISA was performed to detect immunoglobulins specific to the peptides and the designed protein. The assay conditions, including the optimal antigen concentration and serum dilution, were previously established. The evaluation included antigen concentrations ranging from 0.1 to 1.0 μg, pooled sera dilutions from 1:50 to 1:1,000, and peroxidase-conjugated antihuman **IgG** and **IgM** secondary antibody dilutions from 1:5,000 to 1:20,000. Optimal reagent concentrations were determined based on significant differences in absorbance between leprosy patients, household contacts, and healthy subjects. Consequently, ELISA experiments were performed under the optimized conditions. Each peptide and the protein were then tested individually by using samples from the serologic panel. The ELISA plates were coated for 36 h at 4 °C with 1.0 μg/well of soluble peptides and 0.1 μg/well of the multiepitope protein diluted in 50 μL of carbonate buffer (15 mM Na_2_CO_3_; 34 mM NaHCO_3_; pH 9.6). After this step, the procedure was carried out according to a previously described protocol.^29^ The tuberculosis group was tested against only the protein.

### Statistical Analyses

Data were analyzed by using GraphPad Prism 9 for Windows (GraphPad Software, La Jolla, CA, USA). To assess whether the data fit a Gaussian distribution, we used the Shapiro-Wilk normality test. Comparisons between groups were made using the Kruskal–Wallis test, followed by Dunn’s multiple comparison test. Receiver operating characteristic (ROC) analysis was performed to obtain sensitivity, specificity, and accuracy values for **IgG** responses against synthetic peptides and **IgG** and **IgM** responses against the recombinant protein. All serum samples were evaluated in duplicate, with the final result representing the median OD values of these determinations. Cutoff values were determined by the mean plus three times the standard deviation, and intra-assay precision was evaluated using a 10% acceptance threshold for the coefficient of variation.^74,75^ A *p*-value ≤ 0.05 was considered statistically significant. Statistical significance was represented using asterisks according to the following thresholds: * ≤ 0.05, ** ≤ 0.01, *** ≤ 0.001, and **** ≤ 0.0001.

## Supplementary Material



## Notes

Primary sequences and predicted secondary structures of the proteins from which each peptide is derived; positive and negative predictive values (PPV and NPV) of the protein (PDF)

## Abbreviations

HHC: household contacts of leprosy patients
MB: multibacillary leprosy
PB: paucibacillary leprosy
AUC: area under the ROC curve
ROC: receiver operating characteristic
OD: optical density
SE: standard error
CI: confidence interval

## References

[1] de
la Sant M. (2025). Global Leprosy (Hansen Disease) Update, 2024: Beyond
Zero Cases What Elimination of Leprosy Really Means. Wkly. Epidemiol. Rec..

[2] Bhukhan A., Dunn C., Nathoo R. (2023). Case Report of Leprosy in Central
Florida, USA, 2022. Emerging Infect. Dis..

[3] Naidu P., Sharma R., Kanji J. N., Marks V., King A. (2021). Autochthonous
North American Leprosy: A Second Case in Canada. Infect. Dis. Rep..

[4] Health Resources & Services Administration https://www.hrsa.gov/hansens-disease. National Hansen’s Disease (Leprosy) Program. (accessed September 25, 2025).

[5] Young R. A., Mehra V., Sweetser D., Buchanan T., Clark-Curtiss J., Davis R. W., Bloom B. R. (1985). Genes for
the Major Protein Antigens
of the Leprosy Parasite *Mycobacterium Leprae*. Nature.

[6] Lockwood, D. N. ; Lambert, S. Leprosy. In Hunter’s Tropical Medicine and Emerging Infectious Disease; Elsevier, 2013; pp 519–524 10.1016/B978-1-4160-4390-4.00057-6.

[7] Sharma R., Singh P., McCoy R. C., Lenz S. M., Donovan K., Ochoa M. T., Estrada-Garcia I., Silva-Miranda M., Jurado-Santa Cruz F., Balagon M. F., Stryjewska B., Scollard D. M., Pena M. T., Lahiri R., Williams D. L., Truman R. W., Adams L. B. (2020). Isolation of *Mycobacterium
Lepromatosis* and Development of Molecular Diagnostic
Assays to Distinguish *Mycobacterium Leprae* and *M. Lepromatosis*. Clin. Infect. Dis..

[8] Gama R. S., Leite L. A., Colombo L. T., Fraga L. A. de O. (2020). Prospects
for New Leprosy Diagnostic Tools, a Narrative Review Considering ELISA
and PCR Assays. Rev. Soc. Bras Med. Trop.

[9] Martinez A. N., Ribeiro-Alves M., Sarno E. N., Moraes M. O. (2011). Evaluation of QPCR-Based
Assays for Leprosy Diagnosis Directly in Clinical Specimens. PLoS Negl Trop Dis.

[10] Romero C. P., Castro R., do Brasil P. E. A., Pereira D. R., Pinheiro R. O., Toscano C. M., de Oliveira M. R. F. (2022). Accuracy
of Rapid Point-of-Care Serological
Tests for Leprosy Diagnosis: A Systematic Review and Meta-Analysis. Mem. Inst. Oswaldo Cruz.

[11] Lopes-Luz L., Saavedra D. P., Fogaça M. B. T., Bührer-Sékula S., Stefani M. M. de A. (2023). Challenges
and Advances in Serological and Molecular
Tests to Aid Leprosy Diagnosis. Exp. Biol. Med..

[12] de
Alecrin E. S., de Oliveira A. L. G., Guimarães N. S., Lyon S., Martins M. A. P., da Costa Rocha M. O. (2022). Factors
Associated with the Development of Leprosy in Brazilian Contacts:
A Systematic Review. Rev. Inst Med. Trop Sao
Paulo.

[13] Fine P. E. M., Steme J. A. C., Ponnighaus J. M., Bliss L., Saul J., Chihana A., Munthali M., Wamdorff D. K. (1997). Household and Dwelling
Contact as Risk Factors for Leprosy in Northern Malawi. Am. J. Epidemiol.

[14] Butlin C. R., Nicholls P., Bowers B., Quilter E., Alam K., Singh S. (2019). Outcome of Late Healthy
Household Contact Examinations in Leprosy-Affected
Households in Bangladesh. Leprosy Rev..

[15] Gama R. S., Gomides T. A. R., Gama C. F. M., Moreira S. J. M., de
Neves Manta F. S., de Oliveira L. B. P., Marçal P. H. F., Sarno E. N., Moraes M. O., Garcia R. M. G., de
Oliveira Fraga L. A. (2018). High Frequency of *M. Leprae* DNA Detection in Asymptomatic Household Contacts. BMC Infect. Dis..

[16] Duthie M. S., Raychaudhuri R., Tutterrow Y. L., Misquith A., Bowman J., Casey A., Balagon M. F., Maghanoy A., Beltran-Alzate J. C., Romero-Alzate M., Cardona-Castro N., Reed S. G. (2014). A Rapid ELISA for
the Diagnosis of MB Leprosy Based on Complementary Detection of Antibodies
against a Novel Protein-Glycolipid Conjugate. Diagn. Microbiol. Infect. Dis..

[17] Tangye S. G., Ferguson A., Avery D. T., Ma C. S., Hodgkin P. D. (2002). Isotype
Switching by Human B Cells Is Division-Associated and Regulated by
Cytokines. J. Immunol..

[18] Rada E., Duthie M. S., Reed S. G., Aranzazu N., Convit J. (2012). Serologic
Follow-up of IgG Responses against Recombinant Mycobacterial Proteins
ML0405, ML2331 and LID-1 in a Leprosy Hyperendemic Area in Venezuela. Mem. Inst. Oswaldo Cruz.

[19] Soares B. A., Teixeira K. N., de Santana J. F., de Assis B. L. M., Zocatelli-Ribeiro C., Scandelari J. P. S., Thomaz-Soccol V., Machado-de-Ávila R. A., Alvarenga L. M., de Moura J. (2021). Epitope Mapping from *Mycobacterium Leprae* Proteins: Convergent Data from
in Silico and in Vitro Approaches for Serodiagnosis of Leprosy. Mol. Immunol..

[20] Sanchez-Trincado J. L., Gomez-Perosanz M., Reche P. A. (2017). Fundamentals and Methods for T- and
B-Cell Epitope Prediction. J. Immunol. Res..

[21] Davies M. N., Flower D. R. (2007). Harnessing Bioinformatics
to Discover New Vaccines. Drug Discovery Today.

[22] Aguilar-Montes
de Oca S., Montes-de-Oca-Jiménez R., Vázquez-Chagoyán J. C., Barbabosa-Pliego A., Rivadeneira-Barreiro P. E., Zambrano-Rodríguez P. C. (2022). The Use
of Peptides in Veterinary Serodiagnosis of Infectious Diseases: A
Review. Vet. Sci..

[23] Gomara M., Haro I. (2007). Synthetic Peptides for the Immunodiagnosis of Human Diseases. Curr. Med. Chem..

[24] Davies M. N., Flower D. R. (2007). Harnessing bioinformatics
to discover new vaccines. Drug Discovery Today.

[25] Oliveira T. R., Longhi M. T., de Morais Z. M., Romero E. C., Blanco R. M., Kirchgatter K., Vasconcellos S. A., Nascimento A. L. T. O. (2008). Evaluation
of Leptospiral Recombinant Antigens MPL17 and MPL21 for Serological
Diagnosis of Leptospirosis by Enzyme-Linked Immunosorbent Assays. Clin. Vaccine Immunol..

[26] Gonçalves A. A.
M., Ribeiro A. J., Resende C. A. A., Couto C. A. P., Gandra I. B., dos Santos
Barcelos I. C., da Silva J. O., Machado J. M., Silva K. A., Silva L. S., dos Santos M., da Silva Lopes L., de Faria M. T., Pereira S. P., Xavier S. R., Aragão M. M., Candida-Puma M. A., de Oliveira I. C. M., Souza A. A., Nogueira L. M., da Paz M. C., Coelho E. A. F., Giunchetti R. C., de Freitas S. M., Chávez-Fumagalli M. A., Nagem R. A. P., Galdino A. S. (2024). Recombinant
Multiepitope Proteins Expressed in *Escherichia Coli* Cells and Their Potential for Immunodiagnosis. Microb. Cell Fact..

[27] de
Santana J. F., da Silva M. R. B., Picheth G. F., Yamanaka I. B., Fogaça R. L., Thomaz-Soccol V., Machado-de-Avila R. A., Chávez-Olórtegui C., Sierakowski M. R., de Freitas R. A., Alvarenga L. M., de Moura J. (2018). Engineered Biomarkers
for Leprosy Diagnosis Using Labeled and Label-Free Analysis. Talanta.

[28] Alban S. M., de Moura J. F., Thomaz-Soccol V., Sékula S. B., Alvarenga L. M., Mira M. T., Olortegui C. C., Minozzo J. C. (2014). Phage Display and Synthetic Peptides as Promising Biotechnological
Tools for the Serological Diagnosis of Leprosy. PLoS One.

[29] de
Jesus A. C. P., Fraga V. G., Pimenta-Carvalho S. A., Guimarães T. M. P. D., Araújo M. S. S., de Carvalho J. C., Santos M. B., Araújo M. G., Pascoal-Xavier M. A., Lyon S., Ferreira S. R., Arreguin-Campos R., Eersels K., van Grinsven B., Cleij T., Bueno L. L., Bartholomeu D. C., Menezes C. A. da S., Grossi de Oliveira A. L., Fujiwara R. T. (2025). Identifying Promising Peptide Targets for Leprosy Serological
Tests: From Prediction to ELISA. J. Genet. Eng.
Biotechnol..

[30] da
Conceição Oliveira Coel Fabri A., Carvalho A. P. M., Araujo S., Goulart L. R., de Mattos A. M. M., Teixeira H. C., Goulart I. M. B., Duthie M. S., Correa-Oliveira R., Lana F. C. F. (2015). Antigen-Specific
Assessment of the Immunological Status of Various Groups in a Leprosy
Endemic Region. BMC Infect. Dis..

[31] Vidal S. L., De Mattos A. M. M., Menegati L. M., Monteiro T. B. M., Laurindo C. R., Carvalho A. P. M., Teixeira H. C., Coelho A. da C. O. (2019). Testes Sorológicos
Anti-NDO-HSA, Anti-LID-1 e Anti- NDO-LID Em Contatos Domiciliares
de Área Não Endêmica de Hanseníase. HU Rev..

[32] Chen K.-H., Lin C.-Y., Su S.-B., Chen K.-T. (2022). Leprosy: A Review
of Epidemiology, Clinical Diagnosis, and Management. J. Trop. Med..

[33] WHO Expert Committee on leprosy . WHO STUDY GROUP ON TOBACCO PRODUCT REGULATION Report on the Scientific Basis of Tobacco Product Regulation: Fourth Report of a WHO Study Group Introduction. Eighth Report In WHO Technical Report Series; World Health Organization, 2012; pp 1–83. 22953380

[34] Spencer J. S., Dockrell H. M., Kim H. J., Marques M. A. M., Williams D. L., Martins M. V. S. B., Martins M. L. F., Lima M. C. B. S., Sarno E. N., Pereira G. M. B., Matos H., Fonseca L. S., Sampaio E. P., Ottenhoff T. H. M., Geluk A., Cho S.-N., Stoker N. G., Cole S. T., Brennan P. J., Pessolani M. C. V. (2005). Identification
of Specific Proteins and Peptides in *Mycobacterium
Leprae* Suitable for the Selective Diagnosis of Leprosy. J. Immunol..

[35] Cole S. T., Eiglmeier K., Parkhill J., James K. D., Thomson N. R., Wheeler P. R., Honoré N., Garnier T., Churcher C., Harris D., Mungall K., Basham D., Brown D., Chillingworth T., Connor R., Davies R. M., Devlin K., Duthoy S., Feltwell T., Fraser A., Hamlin N., Holroyd S., Hornsby T., Jagels K., Lacroix C., Maclean J., Moule S., Murphy L., Oliver K., Quail M. A., Rajandream M. A., Rutherford K. M., Rutter S., Seeger K., Simon S., Simmonds M., Skelton J., Squares R., Squares S., Stevens K., Taylor K., Whitehead S., Woodward J. R., Barrell B. G. (2001). Massive
Gene Decay in the Leprosy Bacillus. Nature.

[36] Marques M.
A. M., Neves-Ferreira A. G. C., da Silveira E. K. X., Valente R. H., Chapeaurouge A., Perales J., da Silva
Bernardes R., Dobos K. M., Spencer J. S., Brennan P. J., Pessolani M. C. V. (2008). Deciphering the Proteomic Profile of *Mycobacterium Leprae* Cell Envelope. Proteomics.

[37] Geysen H. M., Rodda S. J., Mason T. J., Tribbick G., Schoofs P. G. (1987). Strategies
for Epitope Analysis Using Peptide Synthesis. J. Immunol. Methods.

[38] Atassi M. Z. (1984). Antigenic
Structures of Proteins. Their Determination Has Revealed Important
Aspects of Immune Recognition and Generated Strategies for Synthetic
Mimicking of Protein Binding Sites. Eur. J.
Biochem..

[39] Geluk A., van der Ploeg J., Teles R. O. B., Franken K. L. M. C., Prins C., Drijfhout J. W., Sarno E. N., Sampaio E. P., Ottenhoff T. H. M. (2008). Rational
Combination of Peptides Derived from Different *Mycobacterium
Leprae* Proteins Improves Sensitivity
for Immunodiagnosis of *M. Leprae* Infection. Clin. Vaccine Immunol..

[40] Hoofnagle A. N., Wener M. H. (2009). The Fundamental
Flaws of Immunoassays and Potential
Solutions Using Tandem Mass Spectrometry. J.
Immunol. Methods.

[41] Innocenti G., Andreu-Sánchez S., Hörstke N. V., Elabd H., Barozzi I., Franke A., Manczinger M., Vogl T. (2025). Associations between HLA-II Variation
and Antibody Specificity Are
Predicted by Antigen Properties. Genome Med..

[42] Teles S. F., Silva E. A., de Souza R. M., Tomimori J., Florian M. C., Souza R. O., Marcos E. V. C., de Souza-Santana F. C., Gamba M. A. (2020). Association between NDO-LID and PGL-1 for Leprosy and
Class I and II Human Leukocyte Antigen Alleles in an Indigenous Community
in Southwest Amazon. Braz. J. Infect. Dis..

[43] Freitas A. A., Oliveira R. M., Hungria E. M., Cardoso L. P. V., Sousa A. L. O. M., Costa M. B., Reed S. G., Duthie M. S., Stefani M. M. A. (2015). Alterations
to Antigen-Specific Immune Responses before and after Multidrug Therapy
of Leprosy. Diagn. Microbiol. Infect. Dis..

[44] Freire L. C., Costa S. S., Tedeschi A. L. F., Santos L. M. O., Ribeiro N. R., dos Ries Cruz L., Martins V. T., Galvani N. C., Luiz G. P., de Oliveira M. E., de Ávila R. A.
M., Carvalho A. M. R. S., de Freitas André H. S., Gonçalves D. U., Coelho E. A. F., Roatt B. M., Menezes-Souza D., Duarte M. C. (2025). Synthetic Peptides Derived from Hypothetical Proteins
as Potential Antigens for the Diagnosis of Canine Visceral Leishmaniasis
and Tegumentary Leishmaniasis. Exp. Parasitol..

[45] Moreira G., Maia R., Soares N., Ostolin T., Coura-Vital W., Aguiar-Soares R., Ruiz J., Resende D., de Brito R., Reis A., Roatt B. (2024). Synthetic Peptides Selected by Immunoinformatics
as Potential Tools for the Specific Diagnosis of Canine Visceral Leishmaniasis. Microorganisms.

[46] Gobbo A. R., Bouth R. C., Moraes T. M. P., Pinto P., da Costa P. F., Barreto J. G., Frade M. A. C., Ribeiro-dos-Santos Â. K., de Barros Conde G. A., Duthie M. S., da Silva M. B., Spencer J. S., Salgado C. G. (2022). NDO-BSA,
LID-1, and NDO-LID Antibody
Responses for Infection and RLEP by Quantitative PCR as a Confirmatory
Test for Early Leprosy Diagnosis. Front. Trop.
Dis..

[47] Marçal P. H. F., de Oliveira Fraga L.
A., de Mattos A. M. M., Menegati L., da Conceição Oliveira Coelho A., Pinheiro R. O., Sarno E. N., Duthie M. S., Teixeira H. C. (2018). Utility
of Immunoglobulin Isotypes against LID-1 and NDO-LID for, Particularly
IgG1, Confirming the Diagnosis of Multibacillary Leprosy. Mem. Inst. Oswaldo Cruz.

[48] de
Souza M. M., Netto E. M., Nakatani M., Duthie M. S. (2014). Utility
of Recombinant Proteins LID-1 and PADL in Screening for *Mycobacterium Leprae* Infection and Leprosy. Trans. R Soc. Trop. Med. Hyg..

[49] Cabal A. B. S., Wu T.-Y. (2022). Recombinant Protein
Technology in the Challenging Era
of Coronaviruses. Processes.

[50] Rosano G. L., Ceccarelli E. A. (2014). Recombinant
Protein Expression in *Escherichia
Coli*: Advances and Challenges. Front. Microbiol..

[51] Bhatwa A., Wang W., Hassan Y. I., Abraham N., Li X.-Z., Zhou T. (2021). Challenges Associated
With the Formation of Recombinant Protein Inclusion
Bodies in *Escherichia Coli* and Strategies
to Address Them for Industrial Applications. Front. Bioeng. Biotechnol..

[52] Kost T. A., Condreay J. P., Jarvis D. L. (2005). Baculovirus
as Versatile Vectors
for Protein Expression in Insect and Mammalian Cells. Nat. Biotechnol..

[53] Bill R. M. (2014). Playing
Catch-up with *Escherichia Coli*: Using
Yeast to Increase Success Rates in Recombinant Protein Production
Experiments. Front. Microbiol..

[54] Paul, W. E. Fundamental Immunology, 7th ed.; Lippincott Williams & Wilkins, 2012.

[55] Lakey J. H., Raggett E. M. (1998). Measuring Protein—Protein Interactions. Curr. Opin. Struct. Biol..

[56] Hardie G., van Regenmortel M. H. V. (1975). Immunochemical
Studies of Tobacco
Mosaic Virus—I: Refutation of the Alleged Homogeneous Binding
of Purified Antibody Fragments. Immunochemistry.

[57] Baneyx F., Mujacic M. (2004). Recombinant Protein Folding and Misfolding in *Escherichia Coli*. Nat. Biotechnol..

[58] Sela-Culang I., Kunik V., Ofran Y. (2013). The Structural Basis of Antibody-Antigen
Recognition. Front. Immunol..

[59] Cowan R., Underwood P. A. (1988). Steric Effects in Antibody Reactions with Polyvalent
Antigen. J. Theor. Biol..

[60] Terpe K. (2003). Overview of
Tag Protein Fusions: From Molecular and Biochemical Fundamentals to
Commercial Systems. Appl. Microbiol. Biotechnol..

[61] Chen X., Zaro J. L., Shen W.-C. (2013). Fusion Protein Linkers: Property,
Design and Functionality. Adv. Drug Delivery
Rev..

[62] Brown H., Fastenau A., Penna S., Saunderson P., Klabbers G. (2024). Exploring Active Case Detection Approaches for Leprosy
Diagnosis in Varied Endemic Settings: A Comprehensive Scoping Review. Life.

[63] Barreto J. G., Bisanzio D., Frade M. A. C., Moraes T. M. P., Gobbo A. R., de Souza Guimarães L., da Silva M. B., Vazquez-Prokopec G. M., Spencer J. S., Kitron U., Salgado C. G. (2015). Spatial Epidemiology
and Serologic Cohorts Increase the Early Detection of Leprosy. BMC Infect. Dis..

[64] Ploemacher T., Faber W. R., Menke H., Rutten V., Pieters T. (2020). Reservoirs
and Transmission Routes of Leprosy; A Systematic Review. PLoS Neglected Trop. Dis..

[65] Nicolle D., Ganz J., Mohr M. (2024). A Case of
Zoonotic Domestically Acquired
Hansen Disease (Leprosy) in the State of Georgia. SKIN J. Cutaneous Med..

[66] Aliaga-Samanez A., Deps P. D., Fa J. E., Real R., Guégan J.-F., Oliveira M. A., Pessutti A., Knoop S., Bogoni J. A., Morcatty T. Q., Marques R., Jiménez-García D., Massocato G. F., Desbiez A. L., Kluyber D., El Bizri H. R. (2025). Wildlife
Hunting and the Increased Risk of Leprosy Transmission in the Tropical
Americas: A Pathogeographical Study. Infect.
Dis. Poverty.

[67] Sharma R., Singh P., Loughry W. J., Lockhart J. M., Inman W. B., Duthie M. S., Pena M. T., Marcos L. A., Scollard D. M., Cole S. T., Truman R. W. (2015). Zoonotic Leprosy
in the Southeastern
United States. Emerging Infect. Dis..

[68] da
Silva M. B., Portela J. M., Li W., Jackson M., Gonzalez-Juarrero M., Hidalgo A. S., Belisle J. T., Bouth R. C., Gobbo A. R., Barreto J. G., Minervino A. H. H., Cole S. T., Avanzi C., Busso P., Frade M. A. C., Geluk A., Salgado C. G., Spencer J. S. (2018). Evidence of Zoonotic
Leprosy in Pará, Brazilian Amazon, and Risks Associated with
Human Contact or Consumption of Armadillos. PLoS Neglected Trop. Dis..

[69] Regenmortel, M. H. V. What Is a B-Cell Epitope?. In Epitope Mapping Protocols, Methods in Molecular Biology 2009; pp 3–20 10.1007/978-1-59745-450-6_1. 19377933

[70] Pagniez J., Petitdidier E., Parra-Zuleta O., Pissarra J., Bras-Gonçalves R. (2023). A Systematic
Review of Peptide-Based Serological Tests for the Diagnosis of Leishmaniasis. Parasite.

[71] Wu J., Yang R. (2025). Peptide Biomarkers - An Emerging Diagnostic Tool and
Current Applicable
Assay. Curr. Protein Pept Sci..

[72] Ribeiro A. J., Silva K. A., Lopes L., da S., Resende C. A. A., Couto C. A. P., Gandra I. B., Pereira I. A. G., Barcelos I. C. D. S., Pereira S. P., Xavier S. R., de Sousa Viera Tavares G., Machado J. M., Da Paz M. C., Chávez-Fumagalli M. A., Coelho E. A. F., Giunchetti R. C., Chaves A. T., Dutra W. O., Gonçalves A. A. M., Galdino A. S. (2024). The Use of Peptides
for Immunodiagnosis of Human Chagas Disease. Amino Acids.

[73] Nunes K., e Silva M. A. C., Rodrigues M. R., Lemes R. B., Pezo-Valderrama P., Kimura L., de Sena L. S., Krieger J. E., Catoia
Varela M., de Azevedo L. O., Camargo L. M. A., Ferreira R. G. M., Krieger H., Bortolini M. C., Mill J. G., Sacuena P., Guerreiro J. F., de Souza C. M. B., Veronese F. V., Vianna F. S. L., Comas D., Pereira A. C., Pereira L. V., Hünemeier T. (2025). Admixture’s
Impact on Brazilian Population Evolution and Health. Science.

[74] Linnet K., Bossuyt P. M. M., Moons K. G. M., Reitsma J. B. (2012). Quantifying the
Accuracy of a Diagnostic Test or Marker. Clin.
Chem..

[75] Andreasson U., Perret-Liaudet A., van Waalwijk van Doorn L. J. C., Blennow K., Chiasserini D., Engelborghs S., Fladby T., Genc S., Kruse N., Kuiperij H. B., Kulic L., Lewczuk P., Mollenhauer B., Mroczko B., Parnetti L., Vanmechelen E., Verbeek M. M., Winblad B., Zetterberg H., Koel-Simmelink M., Teunissen C. E. (2015). A Practical Guide to Immunoassay
Method Validation. Front. Neurol..

