# Supplementary material for: A Multiepitope Protein Based on Selected Synthetic Peptides Enables Effective Serological Screening of Exposure to Mycobacterium leprae
Source: ACS Omega. 2025 Dec 22;11(1):522–34. doi: 10.1021/acsomega.5c05816 (PMC12809317; doi:10.1021/acsomega.5c05816)
Supplement: Supplementary file 1 [file ao5c05816_si_001.pdf]

A multiepitope protein based on selected synthetic peptides enables effective serological screening of exposure to *Mycobacterium leprae*

Augusto César Parreiras de Jesus[a,b], Tatyane Martins Cirilo[c], José Bryan da Rocha Rihs[c], Tania Mara Pinto Dabés Guimarães[d], Hayana Ramos Lima[e], Sebastião Rodrigo Ferreira[e], Rocio Arreguin-Campos[b], Cristiane Alves da Silva Menezes[d], Lilian Lacerda Bueno[a,c], Bart van Grinsven[b], Thomas Cleij[b], Ana Laura Grossi de Oliveira[a], Ricardo Toshio Fujiwara[a,c*]

[a]Post-Graduate Program in Infectious Diseases and Tropical Medicine, School of Medicine, Federal University of Minas Gerais, Av. Prof. Alfredo Balena 190, Belo Horizonte, 30130-100, Brazil.

[b]Sensor Engineering Department, Faculty of Science and Engineering, Maastricht University, Duboisdomein 30, Maastricht, 6200MD, The Netherlands.

[c]Post-Graduate Program in Parasitology, Institute of Biological Sciences, Federal University of Minas Gerais, Av. Pres. Antônio Carlos 6627, Belo Horizonte, 31270-901, Brazil.

[d]Department of Clinical and Toxicological Analysis, Faculty of Pharmacy, Federal University of Minas Gerais, Av. Pres. Antônio Carlos 6627, Belo Horizonte, 31270-901, Brazil.

[e]Post-Graduate Program in Health, Environment and Biodiversity, Federal University of Southern Bahia, Av. Pres. Getúlio Vargas, 1732, Teixeira de Freitas, 45996-108, Brazil.

[*]Email: fujiwara@icb.ufmg.br

SUPPORTING INFORMATION

Primary sequences and predicted secondary structures of the proteins from which each peptide is derived. Peptide sequences are highlighted in green in the primary sequences. All figures were generated using PSIPRED (https://bioinf.cs.ucl.ac.uk/psipred/).

Primary sequence S1 – *Full-length protein sequence containing the PEP1 region.*
>WP_010907635.1 septum formation initiator family protein [Mycobacterium leprae]
MSEAKRLDPKRRSPASRPGKAGDSVRGRRSTKPVAKLSVKPSRTTPASSHSGRNSTRM
LTQHVVEPIRQSIIESRERRSDQQLGFTARRAAVLAAVVCVLTLTIAGPVRTYFAQHAEI

EQLAATEATLRRQIADLEQQKGKLADSAYIAARARERLGFVMPGDVPFQVQLPSTAAV
SSQPGGRAAKPANNDPWYTSLWHNIADAPHLPPGAGTPPFSLTPLSTTSGG

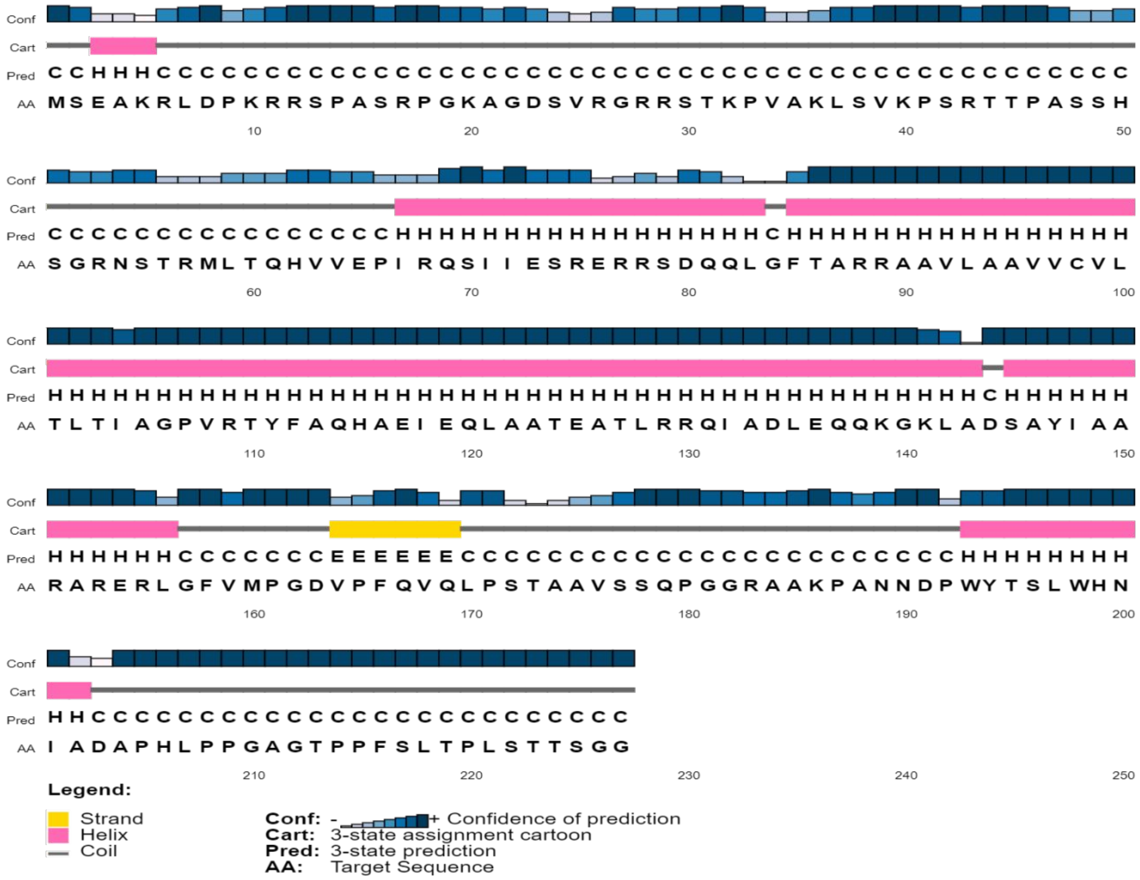

Figure S1 – Cartoon showing the predicted secondary structure of the full-length protein containing PEP1 with confidence level. The schematic cartoon displays strands, helices, and coils in distinct colors representing each structural element. The intensity of blue shading corresponds to the confidence level of the prediction for each residue, with darker shades indicating higher confidence.

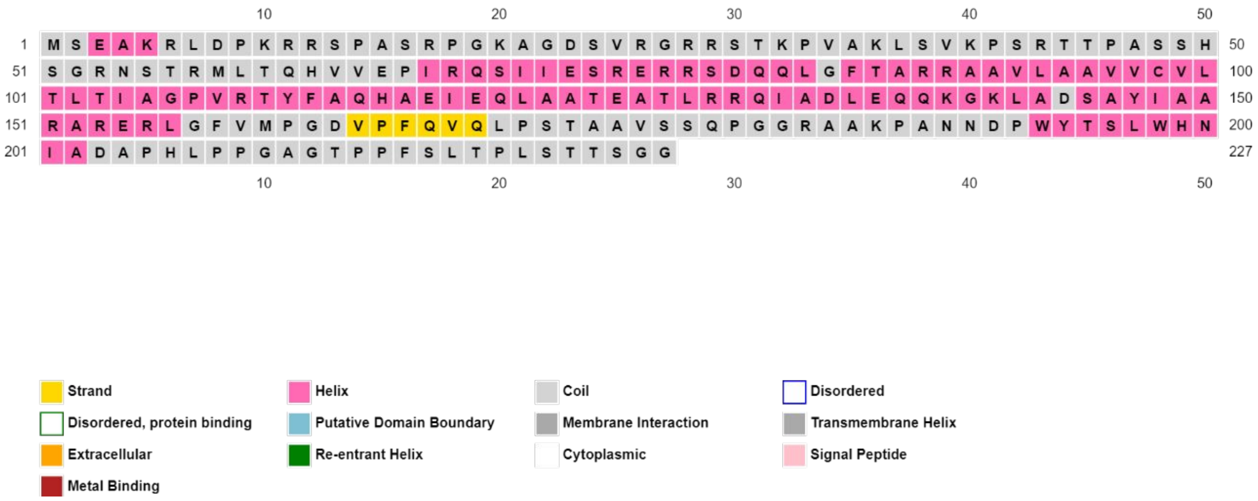

Figure S2 – Sequence plot of the secondary prediction of the full-length protein containing PEP1. Structural elements are color-coded: helices in pink, strands in light yellow, and coil in light grey.

Primary sequence S2 - *Full-length protein sequence containing the PEP2 region.*
>WP_162618330.1 ABC transporter family substrate-binding protein [Mycobacterium leprae]
MSVLISLVDLMLVACTVSTPPAPQSTETPRSSLLPPRITQIIMGIDSIGAGFNPHLLSDLSA
VNAAISALVLPSAFRPATDPNSPTGLRWDMDPTVLVSADVTNQNPFTVTYKIRPEAQW
TDNAPIAADDFWYLWRQMVSQPGVVDPAGYDLITGVQSLEGGKQAVVTFAQPYPAW
RELFSNILPAHIVKDVPGGFAAGLARTLPVTGGQFRVENIDPQRDEILIARNDRYWGPPA
KPGLILFRRAGAPATLADSVRNGDTQLAQVHGGSAAFAQLSAIPGVRTARIVTPRVMQF
TLRANVPKLADIRIRKAILGLLDVDLLAAVGAGSDNTVTLDQAQTRSPSDPGYEPTAPP
ALTTPAAMALLIASGFQIDPITLASPTPNSTASVSTGPLEVIHGRISKDGEQLSLVIGVASN
DPTSVAVANTAADQLRNVGIAATVLALDPVVLYRDALNDNLVDAIVGWHQAGGNLA
TLLASRYGCPALQATEVSTSNTPTASFVGPVGPMQPPNSHSPEPGTLVRAPSNLTGICDH
SIQATIDAALNGSKNINDVITAVEPRLWNMSTVLPILQDTTIVVAGPSVQNISLSGAVPIG
IVGDAGQWTKIGS

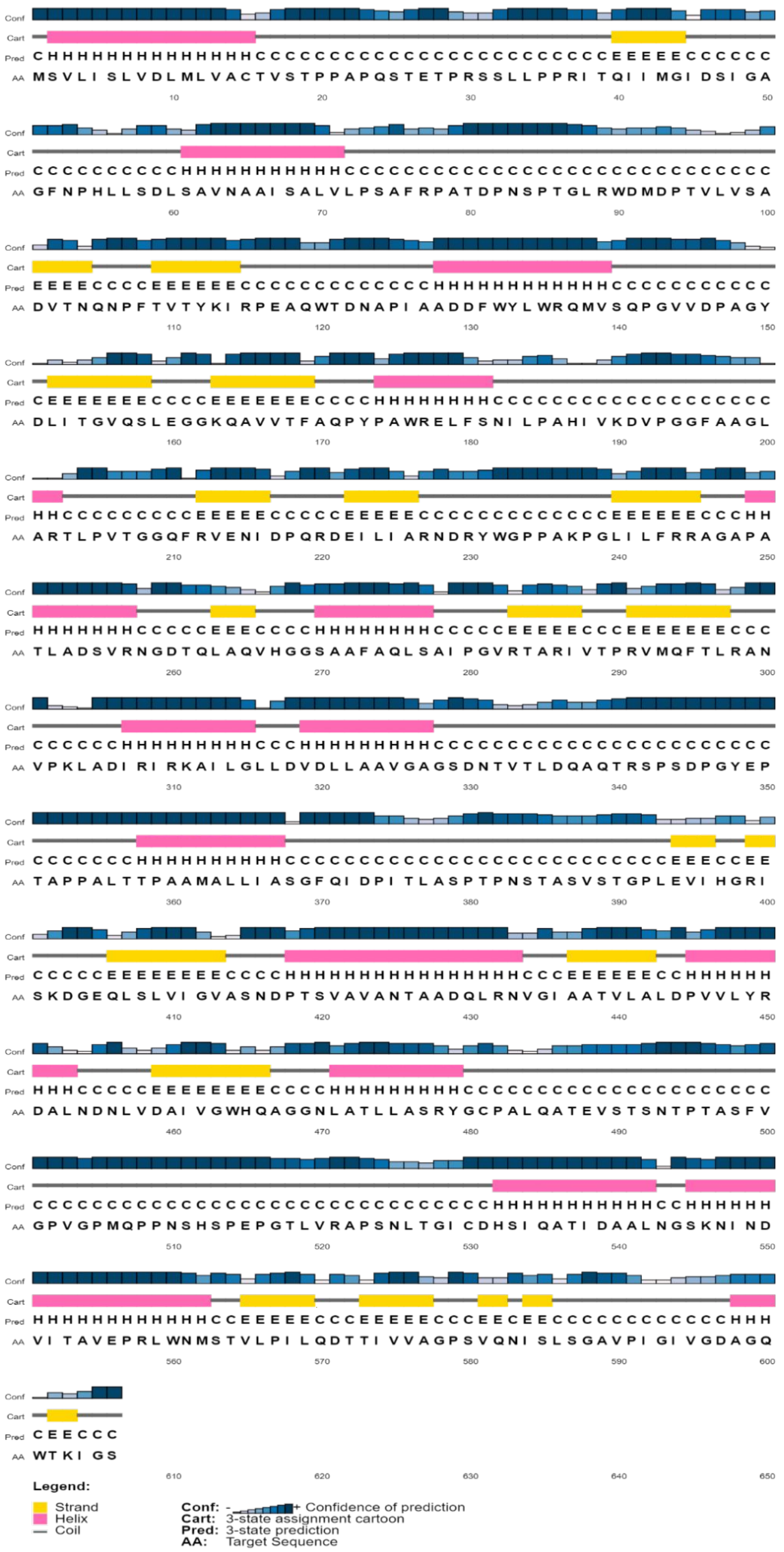
Legend:
Strand
Helix
Coil
Conf: Confidence of prediction
Cart: 3-state assignment cartoon
Pred: 3-state prediction
AA: Target Sequence

Figure S3 - Cartoon showing the predicted secondary structure of the full-length protein containing PEP2 with confidence level. The schematic cartoon displays strands, helices, and coils in distinct colors representing each structural element. The intensity of blue shading corresponds to the confidence level of the prediction for each residue, with darker shades indicating higher confidence.

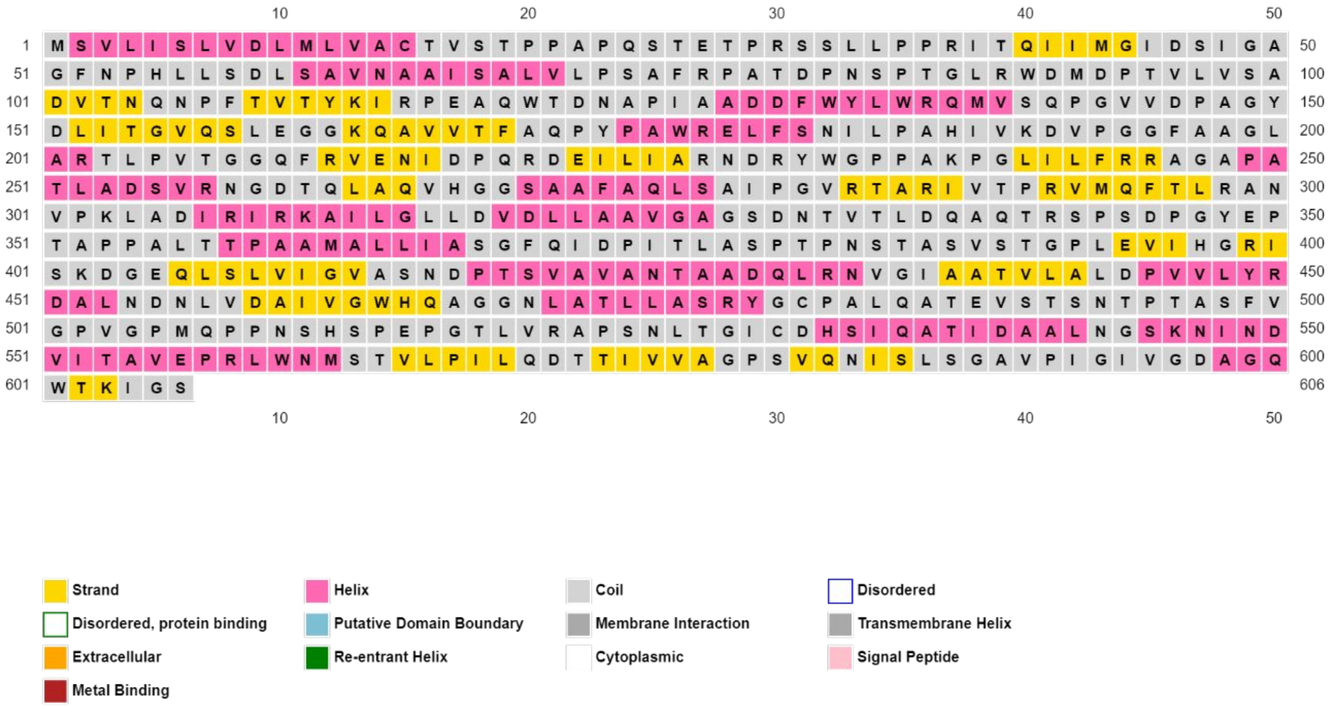

Figure S4 - Sequence plot of the secondary prediction of the full-length protein containing PEP2. Structural elements are color-coded: helices in pink, strands in light yellow, and coil in light grey.

Primary sequence S3 - *Fragment of the protein sequence, comprising residues 1053 to 1871, which includes the PEP3 region.*

>WP_010908856.1 type I polyketide synthase [Mycobacterium leprae]
MTASIGSEADLCHWLVNYLVTNIGCTPDEVDLNLSLTDLGMSSRDAVVLSGELTDLLG
KTVSPIDFWEHPTINALAAYLIAPAPDPESESAPRRSVQGALEEPIAVIGMGCRFPGGISG
PEALWQFLCDRRSSIGQVPNERWELFDDGSPEVKALLARTTRWGSFLEDIDAFDSEFFEI
SPSEADKMDPQQRLLLEVAWEALEHAGISSNSLRRSQTGVFAGSCLSEYGAIASTDLSQ
VDGWSNTGGAMSIIANRLSYFLDLRGPSVAVDTACSSSLVAIHLACQSLRTADANLAIA
AGVNLLLSPAVFRGFDQVGALSPTGCCRAFDAAADGFVRGEGAGVVVLKRLTDAQRD
GDRVLAIIRGSAVNQDGRSNGLMAPNPAAQVAVLRTAYANAAMPPTAIDYVETHGTG
TLLGDPIEARALGAVLGRGRAEDSPLLIGAVKTNLGHLEAAAGIAGFIKTVLAVQRGQI
PPNQRFESPNLHIPFAELRMKVVDKHMEWPDTGHLRRAAVSSFGFGGTNAHVVIEQGP
ELTPVTECSSNTAVSTLVVTGKTASRVAAMAGMLADWVEGPGAEVALADVAHTLNH
HRSRHAKFGTVVARDRIQAVAGLRALAAGKQAPGVVGQQDGTPGSGTVFVYSGRGSQ

WAGMGRQLLADEPAFTAAVAELEPVFVVHAGFSLHDVLANGKELVGIEQIQLGLIGM
QLTLTELWRSYGVQPDLVIGHSLGEVAAAVVAGALTAAEGLRVTATRSRLMAPLSGQ
GGMALLELDAVETEALIVDYSQVTLAIYNSPRQTVIAGPTEQIDELIDRVRAQNRFASRV
NIEVAPHNPAMDALQPQMRSELADVAPRTPTIPILSTTYADLGSCPVFDAQHWATNMR
NPVHFQQAIMTAGTDHRTFIEISAHPLLTQAITDTLHGTRCISIGTLQRDADDTVTFHTNL
NNVHTVHPPHTPHPAEPHVTIPSTPWQHTRHWIPRKRSVNSIGLAPQVGTLLGQHTAVS
GILPIHLWQARLAPQAKPYRGWHRVHGVEVVPASIVLRTILCAAAELGYSTLFEIRFEQP
IFANLPRLIQVVVDNQLISLASSPATETPQHRWTRHVTAQLSSASARSAVPSDHPDQING
HLEVISDPIPDVASLLALCGVDGLPFEWSVKSWAKKTQQSTSRTVEIEFPDTLPDGAIAP
LLDAAILIPALADVTETQFYVPASIKQVWLGDTATGPCGAVTLNRTACDDDGITVDVTV
ADGSGVPLALMRSLRYRALDLDNAQQDYSTVFVESHVDALTDARNFVHAIDWQPRTD
LDCSNTPVASSGSVAVIGDARAGFGLLLKEAGYTLAAPADGVSEVRYVVYVADPHPAT
TAETDVDFAVRITAEISDLVRALAKREPGKPVALWIVTRGVYESVASSALRQSFLWGFG
GVIAAEHPEMWGGLVDLAASALGADRDDILDQFGPTLASLLQTPTRSIVVLRDGVVLA
PTLVPIRSEPVRKSLQCKSDGAYLITGGMGALGLLMADWLADRGARRLVLMGRTPLPP
RRDWELDTLDAGLRQKIDAICALEMRGVTVEAVAVDVGHHDDVQALLAKRDRDGAA
PIRGIIHAAGVTNDQLVTNMTDDPVRQVMWPKIAGCQVLHEAFPPGSVDFFYLTSSAA
GIFGIPGQGSYAAANSYLDALARTRRQQGCHTMSIDWVAWRGLGFAANAQIVSDELQ
RMGSRDITPSEAFTAWEYVDAYDVAQAVVLPVPRSYGSAGSTAGDSYLVPARNWSQM
EAAEVRKELESGLRTIIAAELRVAEAELDTDRPFAELGLNSLMAMAIRREAEQFIGIELS
AIMLFNHPTVALLAAYLTKVVAPQHDSQGDEMAALSASAGGVLDSLFDRIESTSTEAE
GLV

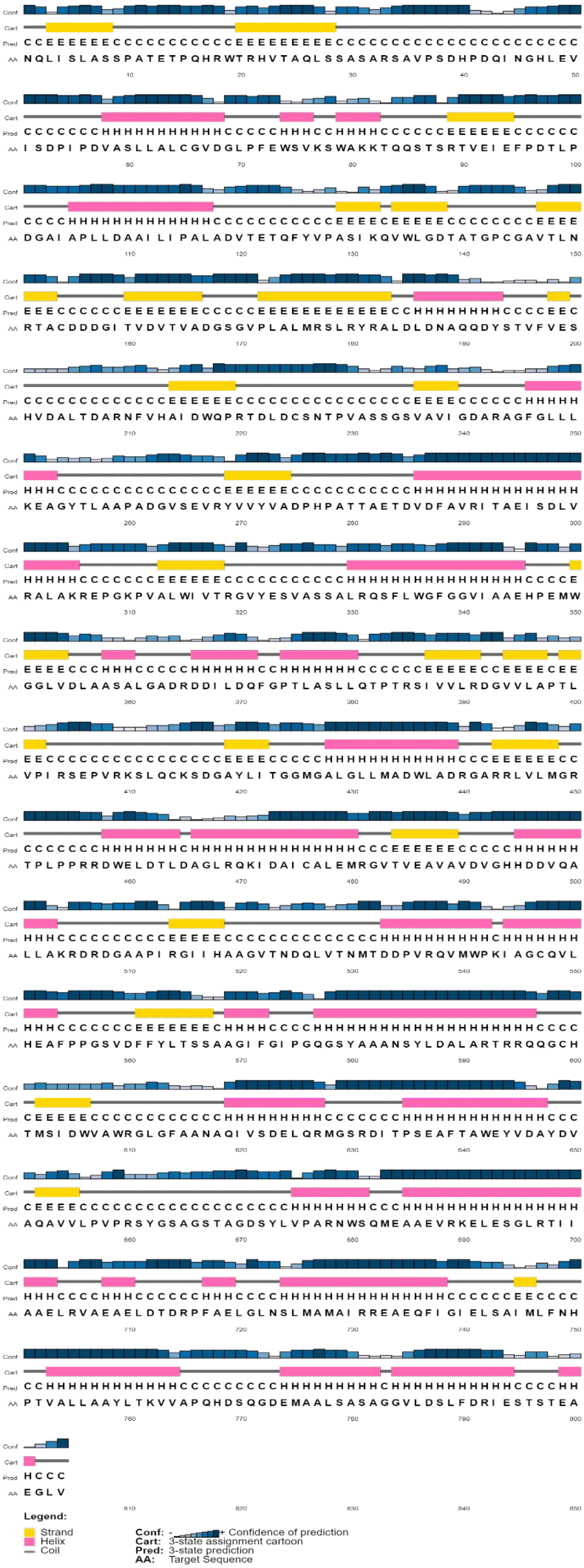
Pred CCEEEEEECCCCCCCCCCEEEEEEEEECCCCCCCCCCCCCCCCCCCCCCC
AA NQLISLASSPATETPQHRWTRHVTAQLSSASARSAVPSDHPDQINGHLEV
Pred CCCCCCCHHHHHHHHHHHCCCCCHHHCCHHHHCCCCCCEEEEEEECCCCCC
AA ISDPIPDVASLLALCGVDGLPFEWSVKSWAKKTQQSTSRTVEIEFPDTLP
Pred CCCCHHHHHHHHHHHHHCCCCCCCCCCCEEEEECEEEEEECCCCCCCCEEEE
AA DGAIAPLLDAAILIPALADVTETQFYVPASIKQVWLGDTATGPCGAVTLN
Pred EEECCCCCCEEEEEEEECCCCCEEEEEEEEEEEEEECCHHHHHHHHCCCCEEC
AA RTACDDDGITVDVTVADGSGVPLALMRSLRYRALDLDNAQQDYSTVFVES
Pred CCCCCCCCCCCCCEEEEEECCCCCCCCCCCCCCCCCCCEEEECCCCCCHHHHH
AA HVDALTDARNFVHAIDWQPRTDLDCSNTPVASSGSVAVIGDARAGFGLLL
Pred HHHCCCCCCCCCCCCCCCEEEEEECCCCCCCCCCCHHHHHHHHHHHHHHH
AA KEAGYTLAAPADGVSEVRYVVYVADPHPATTAETDVDFAVRITAEISDLV
Pred HHHHHCCCCCCCEEEEEECCCCCCCCCCCHHHHHHHHHHHHHHHHHCCCCE
AA RALAKREPGKPVALWIVTRGVYESVASSALRQSFLWGFGGVIAAEHPEMW
Pred EEEECCCHHHCCCCCHHHHHHCCHHHHHHHCCCCCCEEEEECCEEEEECEE
AA GGLVDLAASALGADRDDILDQFGPTLASLLQTPTRSIVVLRDGVVLAPTL
Pred EECCCCCCCCCCCCCCCCEEEECCCCCHHHHHHHHHHHHCCCEEEEEEECC
AA VPIRSEPVRKSLQCKSDGAYLITGGMGALGLLMADWLADRGARRLVLMGR
Pred CCCCCCCHHHHHHHCHHHHHHHHHHHHHHHCCCEEEEEECCCCCHHHHHH
AA TPLPPRRDWELDTLDAGLRQKIDAICALEMRGVTVEAVAVDVGHHDDVQA
Pred HHHCCCCCCCCCCEEEEECCCCCCCCCCCCCCHHHHHHHHHCHHHHHHH
AA LLAKRDRDGAAPIRGIIHAAGVTNDQLVTNMTDDPVRQVMWPKIAGCQVL
Pred HHHCCCCCCCEEEEEEEECHHHHCCCCHHHHHHHHHHHHHHHHHHHHHCCCC
AA HEAFPPGSVDFFYLTSSAAGIFGIPGQGSYAAANSYLDALARTRRQQGCH
Pred CEEEEECCCCCCCCCCCCHHHHHHHHHCCCCCCCHHHHHHHHHHHHHHCCC
AA TMSIDWVAWRGLGFAANAQIVSDELQRMGSRDITPSEAFTAWEYVDAYDV
Pred CEEEECCCCCCCCCCCCCCCCCCCCHHHHHHHCCCHHHHHHHHHHHHHHHHH
AA AQAVVLPVPRSYGSAGSTAGDSYLVPARNWSQMEAAEVRKELESGLRTII
Pred HHHCCCCHHHCCCCCCHHHCCCCHHHHHHHHHHHHHHHCCCCCCEECCCCC
AA AAELRVAEAELDTDRPFAELGLNSLMAMAIRREAEQFIGIELSAIMLFNH
Pred CCHHHHHHHHHHHHCCCCCCCCCHHHHHHHHHCHHHHHHHHHHHCCCCHH
AA PTVALLAAYLTKVVAPQHDSQGDEMAALSASAGGVLDSLFDRIESTSTEA
Pred HCCC
AA EGLV
Legend:
Strand
Helix
Coil
Conf: - + Confidence of prediction
Cart: 3-state assignment cartoon
Pred: 3-state prediction
AA: Target Sequence

Figure S5 - Cartoon showing the predicted secondary structure of the protein containing PEP3 with confidence level. The schematic cartoon displays strands, helices, and coils in distinct colors representing each structural element. The intensity of blue shading corresponds to the confidence level of the prediction for each residue, with darker shades indicating higher confidence.

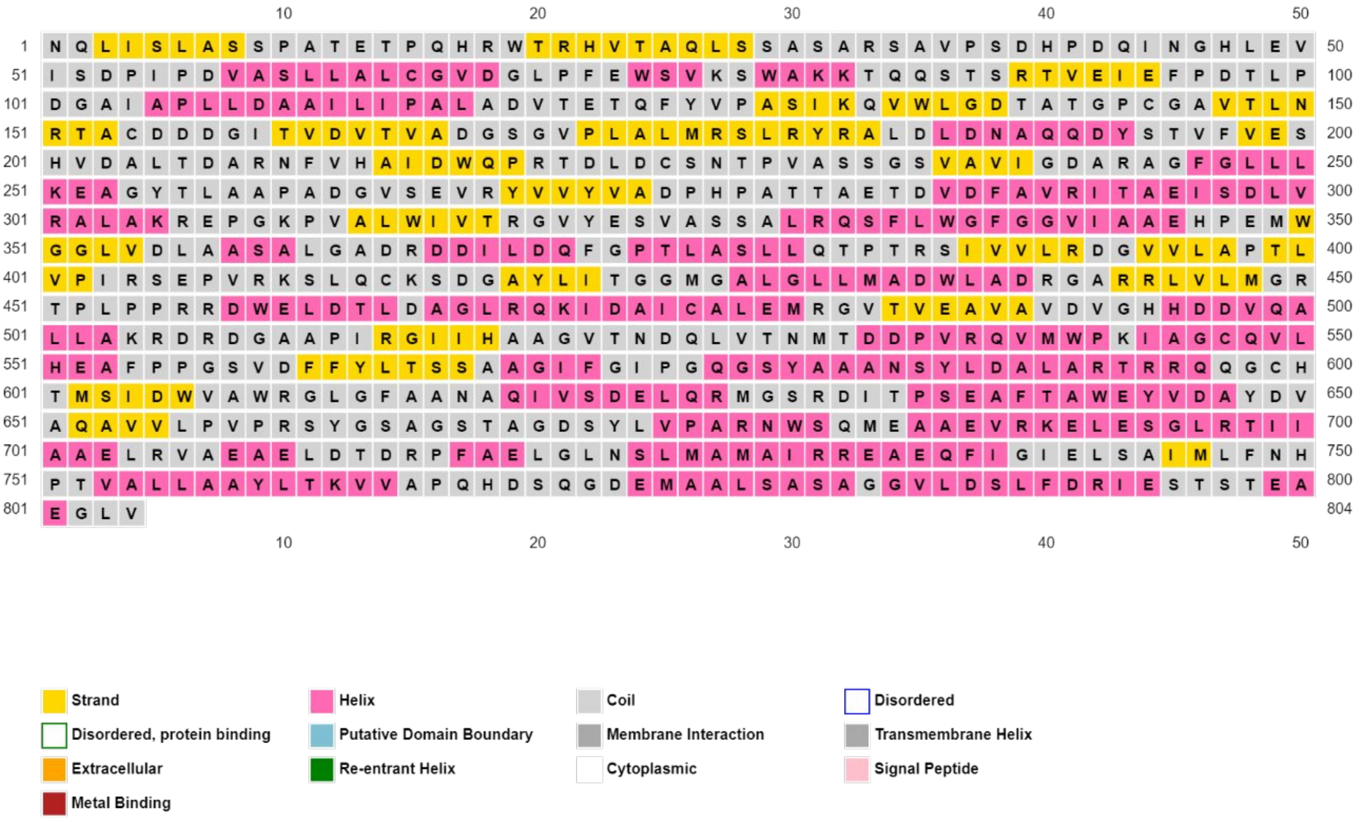

Figure S6 - Sequence plot of the secondary prediction of the protein containing PEP3. Structural elements are color-coded: helices in pink, strands in light yellow, and coil in light grey.

Primary sequence S4 - *Full-length protein sequence containing the PEP4 region.*
>AWV48171.1 hypothetical protein DIJ64_09190 [Mycobacterium leprae]
MLGPSIGAYPDRHDSSDKIEASLRHLPRSSEAAGDRAGRGQDRSSSSRIDLTVASLCARR
PRRWGAGATVSRG

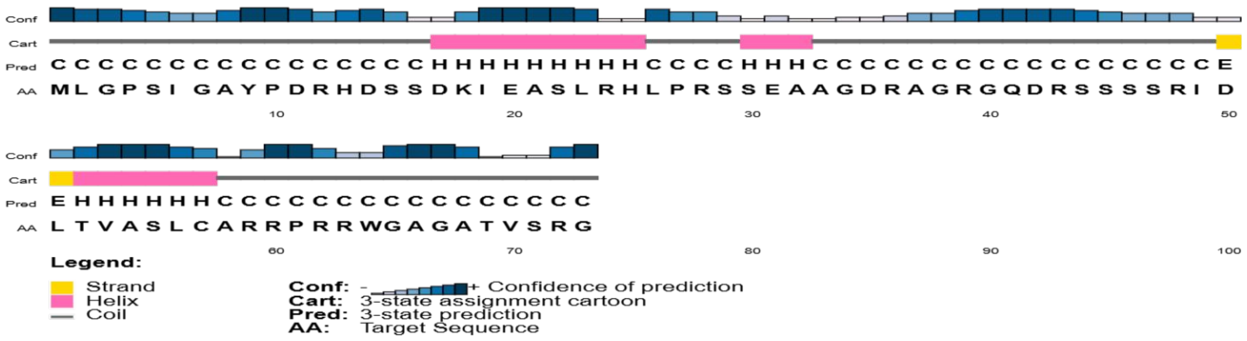

Figure S7 - Cartoon showing the predicted secondary structure of the full-length protein containing PEP4 with confidence level. The schematic cartoon displays strands, helices, and coils in distinct colors representing each structural element. The intensity of blue shading corresponds to the confidence level of the prediction for each residue, with darker shades indicating higher confidence.

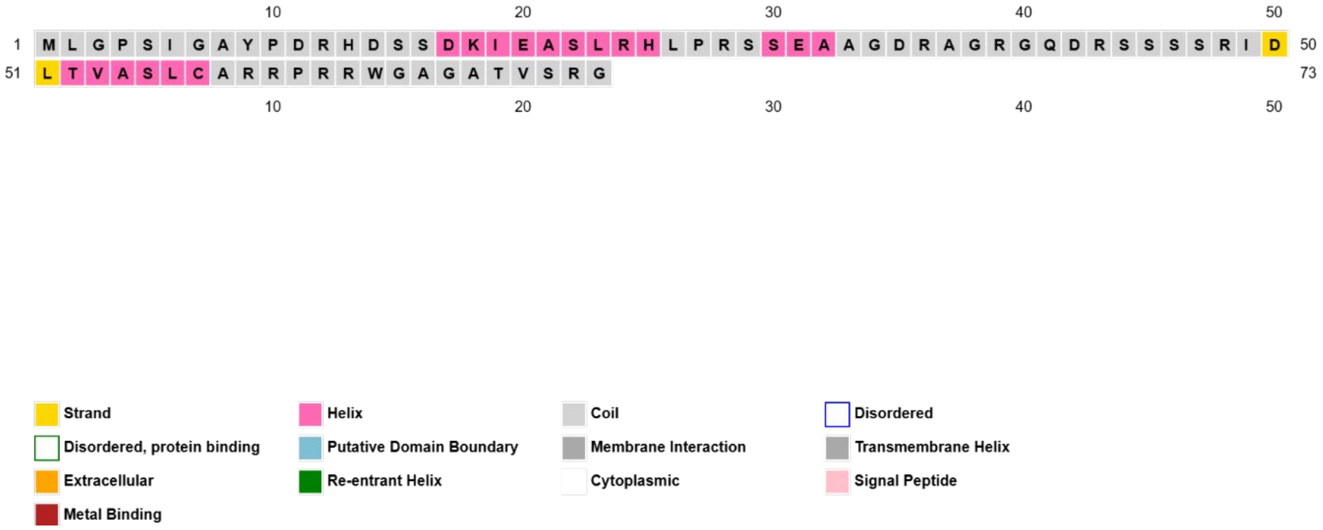

Figure S8 - Sequence plot of the secondary prediction of the full-length protein containing PEP4. Structural elements are color-coded: helices in pink, strands in light yellow, and coil in light grey.

Primary sequence S5 - *Full-length protein sequence containing the PEP5 region.*
>WP_010908290.1 NAD kinase [Mycobacterium leprae]
MTPRKAAQRTVLLVVHTGRDEATETARRVKKIVGDNGIALRVLSAEAVDRGSLHLAL
DNMRAMGVDIEVVDADPHVAQGCELVLVLGGDGTFLRAAELARTARIPVLGVNLGRI
GFLAEAEAEAIDVVLEHVIARSYRVEERLTLDIVVRQGGNIIDQGWALNEASLEKGPRL
GVLGVVVEIEGRPVSTFGCDGVLVSTPTGSTAYAFSAGGPVLWPDLEAILVVPNNAHA
LFGRPMVTSPDATVAIELEANGNDALVFCDGRREMIIPAGGRLEVTRCATPVKWARLD
SAPFTDRLVSKFRLPVTGWRGK

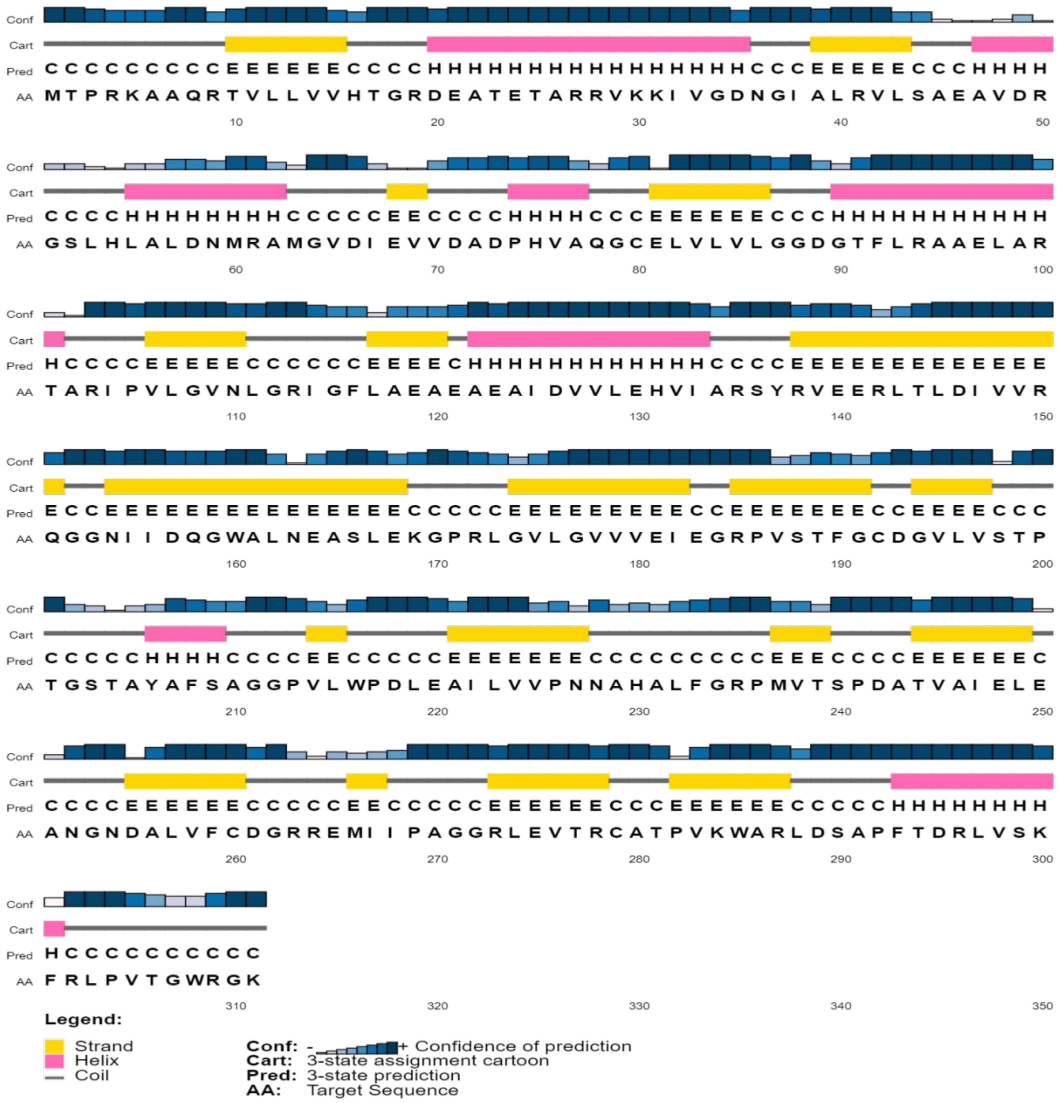

Figure S9 - Cartoon showing the predicted secondary structure of the full-length protein containing PEP5 with confidence level. The schematic cartoon displays strands, helices, and coils in distinct colors representing each structural element. The intensity of blue shading corresponds to the confidence level of the prediction for each residue, with darker shades indicating higher confidence.

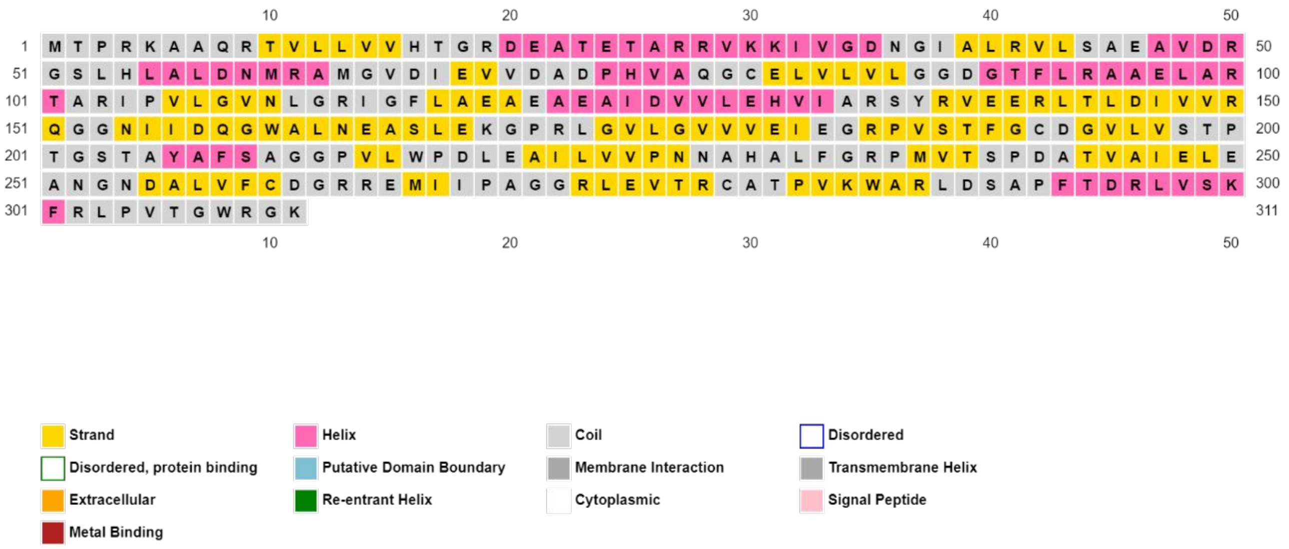

Figure S10 - Sequence plot of the secondary prediction of the full-length protein containing PEP5. Structural elements are color-coded: helices in pink, strands in light yellow, and coil in light grey.

Table S1. Positive and negative predictive values (PPV and NPV) of the protein, calculated using clinical diagnosis as the reference for patients and epidemiological classification for contacts. *PPV, positive predictive value; NPV, negative predictive value;*

Population	IgM		IgG	
	PPV (%)	NPV (%)	PPV (%)	NPV (%)
Patients	86.4	29.1	89.7	35.6
Contacts	85.2	48.3	88.7	54.2
